# Genome-wide histone modification profiling of inner cell mass and trophectoderm of bovine blastocysts by RAT-ChIP

**DOI:** 10.1371/journal.pone.0225801

**Published:** 2019-11-25

**Authors:** Tõnis Org, Kati Hensen, Rita Kreevan, Elina Mark, Olav Sarv, Reidar Andreson, Ülle Jaakma, Andres Salumets, Ants Kurg

**Affiliations:** 1 Department of Biotechnology, Institute of Molecular and Cell Biology, University of Tartu, Tartu, Estonia; 2 Chair of Animal Breeding and Biotechnology, Estonian University of Life Sciences, Tartu, Estonia; 3 Competence Centre on Health Technologies, Tartu, Estonia; 4 Department of Bioinformatics, Institute of Molecular and Cell Biology, University of Tartu, Tartu, Estonia; 5 Institute of Genomics, University of Tartu, Tartu, Estonia; 6 Department of Obstetrics and Gynaecology, Institute of Clinical Medicine, University of Tartu, Tartu, Estonia; 7 Institute of Biomedicine and Translational Medicine, University of Tartu, Tartu, Estonia; 8 Department of Obstetrics and Gynaecology, University of Helsinki and Helsinki University Hospital, Helsinki, Finland; Hirosaki University Graduate School of Medicine, JAPAN

## Abstract

Chromatin immunoprecipitation coupled with next-generation sequencing (ChIP-seq) has revolutionized our understanding of chromatin-related biological processes. The method, however, requires thousands of cells and has therefore limited applications in situations where cell numbers are limited. Here we describe a novel method called Restriction Assisted Tagmentation Chromatin Immunoprecipitation (RAT-ChIP) that enables global histone modification profiling from as few as 100 cells. The method is simple, cost-effective and takes a single day to complete. We demonstrate the sensitivity of the method by deriving the first genome-wide maps of histone H3K4me3 and H3K27me3 modifications of inner cell mass and trophectoderm of bovine blastocyst stage embryos.

## Introduction

The development and functioning of a multicellular organism are determined by how the genetic information, encoded in its genomic DNA sequence, is utilized by individual cells. In eukaryotic cells, DNA is packed into chromatin using proteins of which histones are the most abundant. Each cell type has its specific chromatin structure, which is dynamic and can be remodeled to regulate gene expression, DNA repair and cell division. Our understanding of these chromatin-related processes has improved vastly over the past years thanks to the advances in DNA sequencing technologies. Chromatin immunoprecipitation (ChIP) has been the method of choice to study the location of DNA bound proteins for years [1]. Coupling ChIP with deep sequencing (ChIP-seq) has enabled to determine the localization of chromatin-bound proteins at a genome-wide level [2]. For example, it has helped to identify that different histone post-translational modifications are associated with different genomic features and transcriptional states, helping to explain how cell type specific gene regulation is achieved [3,4].

One of the limitations of ChIP-seq method is that it usually requires a large number of cells. In a typical experiment, several million cells are used, which can be a limiting factor when working with samples where cell numbers are restricted, such as highly purified rare cell populations or early developmental stages. Recent advances in technologies have made it possible to develop more sensitive ChIP-seq methods (S1 Table) [5–19], however, many of these methods are complex, laborious, require specific apparatus or reagents, or are not sensitive enough. With these limitations in mind, we aimed to develop a novel ChIP-method for a limited number of cells that would be sensitive but also robust so it could be used without the need for special equipment and readily available reagents.

A typical ChIP-seq experiment consists of several experimental steps to produce a library that can be sequenced using massively parallel sequencing. These steps include fixing, cell lysis, chromatin fragmentation, immunoprecipitation, decrosslinking, DNA purification, and sequencing library preparation followed by sequencing. There are many issues that arise when working with a low number of cells of which the loss of material is the most prominent. In the published protocols the reduction of material loss has been achieved through the use of different carriers that mimic more material [7,12][19] or with indexing first and then pool the samples technique to obtain more material for subsequent steps [9,14,15]. Another option is to minimize the number of experimental steps, such as centrifugations and material transfer from one tube to another, where a material loss is expected. In addition, it is desirable to downscale the sample volumes to account for the reduction in the amount of input material. This can be relatively easily achieved when the sonication step is replaced with enzymatic DNA digestion, as using standard equipment, sonication cannot be done effectively in a small volume.

There are many instances where more sensitive ChIP-seq methods could be useful but the most obvious is studying early development, like mammalian embryo preimplantation development, as the number of available cells in these study samples is especially low. The cellular changes that take place during development are remarkable both at the molecular and phenotypic level. Understanding the molecular basis for differentiation it is not only fascinating but it also holds great promise to advance reproductive medicine and the generation of desired cell types for regenerative medicine. Recently, the first histone modification landscapes of early mouse development were reported [16,17,20]. In addition to using more sensitive ChIP assays, a pool from large number of embryos was still needed in order to obtain a sufficient amount of cells. Therefore, although substantial advancements have been made both in sensitivity and simplicity of the ChIP-seq methods, there is still room for improvement. This is in particular important in the context of studying human embryonic development as many legal, ethical and technical issues come to play. To overcome some of these issues, bovine can be used as a model as its embryogenesis is more similar to the human compared to other common model organisms. Previously, we have used bovine to study chromosomal instability in early embryos and shown it to be a good model [21]. Here, by using a novel RAT-ChIP method we derive the first genome-wide histone H3K4me3 and H3K27me3 profiles of inner cell mass (ICM) and trophectoderm (TE) of blastocyst stage bovine embryos, being the two major cell lineages that develop into embryonic and extraembryonic tissues, respectively. Combining this epigenetics data with published expression profiling data serves as a resource to provide insights into the gene expression regulation of early bovine development and paves a way to new studies, for example with a somatic cell nuclear transfer (SCNT), e.g. cloned embryos where epigenetics has thought to act as a major roadblock of nuclear reprogramming.

## Materials and methods

### Cell lines

Human K562 and H1299 cells were grown in IMDM and DMEM (both from Naxo) respectively, supplemented with 10% of FBS and penicillin/streptomycin (Naxo) in the presence of 5% CO_2_ at 37°C.

### Oocyte collection and *in vitro* maturation

All chemicals used for *in vitro* embryo production were purchased from IVF Limited T/A IVF Bioscience. Slaughterhouse derived ovaries were transported to the laboratory in a 0.9% sterile NaCl solution within 4 h after slaughter at approximately 32–37°C and washed twice in a 0.9% NaCl solution. Using a vacuum pump (Minitüb GmbH), cumulus oocyte complexes (COC) from follicles with a diameter of 2-8mm were aspirated. Grade 1 COCs were washed and matured in groups of 50 in 500μl of *in vitro* maturation medium in four-well plates (Nunc). Oocytes were incubated at 38.5°C with humidified 5% CO_2_ in air for 22–24 h.

### *In vitro* fertilization

Frozen-thawed semen from a Holstein bull ZIARD (id EE 13993023) was used to fertilize the matured oocytes. Oocytes and sperm were co-incubated in groups of 50 in 500μl of BO-IVM media in four-well plates (Nunc) at 38.5°C with humidified 5% CO_2_ in air for 22–24 h.

### *In vitro* cultivation

Zygotes were individually cultured in 60μl droplets BO-IVC media for 8 days at 38.5°C, 5% CO_2_, 5% O_2_ and 90% N_2_ with maximum humidity. Embryos having reached blastocyst stage by day 8 were collected and used for laser-assisted microdissection.

### Laser-assisted microdissection to obtain ICM and TE fractions

Integra 3 micromanipulator (Research Instruments Limited) equipped with Saturn 5 Active^™^ laser system was used to manually separate bovine blastocysts into ICM and TE fractions (of note–while manual dissection achieves to get pure populations of TE, small fraction of TE cells remain associated with the separated ICM mass). Separated fractions from three blastocysts were pooled and used for subsequent RAT-ChIP-seq experiments.

### RAT-ChIP-(seq)

For a single immunoprecipitation 1μl ProtG Dynabeads (Thermo Fisher Scientific) were bound with 0.25μg of corresponding antibody (H3K4me3 (07–473, Millipore), H3K27me3 (07–449, Millipore)) or H3 (C15200011, Diagenode) in 5μl of complete immunoprecipitation (IP) buffer (20mM Tris HCl pH 7.4, 2mM EDTA, 150mM NaCl, 0.1% Triton X-100) at RT using standard 0.2ml Eppendorf tubes with end-over end mixing (30 rpm) for 2 h followed by two washes using 50μl of IP buffer. All washes were performed by gently pipetting the beads 10 times up and down. Magnetic beads were captured by 1-minute incubation on a magnetic stand (Diagenode). Finally, beads were suspended in the original amount of IP buffer (1μl per IP).

The density of cultured K562 or H1299 cells was determined using haemocytometer. Cells were spun down and resuspended in PBS at a density 100 or 1,000 cells per 0.5μl. Subsequently 0.5μl of lysis-restriction mix (1μl FD (FastDigest) buffer combined with 3.75μl of 2x nuclear lysis buffer (20mM Tris HCl, pH 7.4, 20mM NaCl, 6mM MgCl_2_, 0.2% NP-40)) and 0.25μl 4x restriction enzyme mix (AluI #FD0014, SaqAI#FD2174, HinfI#FD0804, MvaI#FD0554, all FastDigest enzymes from Thermo Scientific in equal amounts) was added to the cells and incubated 15 min on ice and thereafter 5 min at 37°C. Next, 1μl of 0.2% NaDOC, 0.2% TritonX-100 with protease inhibitors was added to the samples and incubated 15 min on ice, vortexed for 30 sec, after which 8μl of IP buffer (20mM Tris HCl, pH 7.4, 150mM NaCl, 2mM EDTA and 1% TritonX-100) and 1μl of ProtG Dynabeads (Thermo Fisher Scientific) prebound with corresponding antibody was added to the samples. Chromatin immunoprecipitation was performed at 4°C for four hours with end-over end mixing (30 rpm). After IP, beads were washed twice with 100μl of following buffers: low salt washing buffer (0.1% SDS, 1% Triton X-100, 2mM EDTA, 20mM Tris HCl (pH 8.0), 150mM NaCl), high salt washing buffer (0.1% SDS, 1% Triton X-100, 2mM EDTA, 20mM Tris HCl (pH 8.0), 500mM NaCl), IP buffer and 20mM Tris HCl, pH 7.4, as described above. To reduce background the beads were carried over to a new tube during the last wash. Multichannel pipet was used for processing multiple samples simultaneously.

Tagging was performed by resuspending the beads in 2.5μl of transposase mix (prepared by mixing 5μl of 2x DNA tagment buffer with 4μl mQ and 1μl Transposase) (Illumina Nextera kit) and incubation of 1 min at 37°C. Beads were washed once with 100μl of low salt washing buffer, once with 20mM Tris HCl pH 7.4, as described above, and resuspended in 5μl of 20mM Tris HCl, pH 7.4. 16 cycles of PCR were performed using bead bound DNA as a template by mixing 5μl of beads with 2,5μl of 5μM forward and reverse primers from the ATAC-seq protocol [22] and 10μl of 2x NEBNext PCR master mix (New England Biolabs). The following PCR program was used: (72°C 5 min, 98°C 2 min, 98°C 10 sec, 63°C 10 sec, 72°C 1 min, repeat steps 3–5 15 times, hold at 4°C). PCR products were purified with Agencourt RNA XP magnetic beads (Beckman Coulter), eluted in 10μl of Tris HCl pH 7.4 followed by quality control and DNA quantification using NanoDrop, Qubit (Thermo Fisher Scientific) and TapeStation (Agilent). The resulting library was subjected to 50 or 75bp paired-end Illumina sequencing using either HiSeq2500 or NextSeq platforms. Alternatively, the library was analyzed with q-RT-PCR using Applied Biosystems 7900HT real-time qPCR machine, HOT FIREPol^®^ EvaGreen^®^ qPCR Mix Plus (Solis BioDyne) and following primers: GAPDH_F CCCGTCCTTGACTCCCTAG, GAPDH_R CTGGTTCAACTGGGCACG; VPS29_F TCGCTACTTCCTGTTCTGCA, VPS29_R GATAGGGGCACGGTCCTC; ZNF7_F TACTGTTTCCTCGCCAGCTC, ZNF7_R GAGGCAAAGGAGACAAAGCA; Neg_cntrl_F CAAATGTGGTCACTAAGGCAAC, Neg_cntrl_R GTGACTCTCCTGGACCAACA.

RAT-ChIP-seq with bovine blastocysts was performed as described above except that after dissection the cells were collected in 3μl of embryo medium. Lysis/restriction buffer was prepared by combining 4.75μl of 10x NL (100mM Tris HCl, pH 7.4, 100mM NaCl, 30mM MgCl_2_) buffer, 4.75μl of 10x FastDigest buffer and 0.5μl of mix of 4x restriction endonucleases. 0.75μl of lysis/restriction buffer was added to 3μl of cells and incubated 15 min on ice and 5 min at 37°C. Next, 1μl of mix of 0.5% NaDOC, 0.5% TritonX100 with protease inhibitor cocktail was added to the samples and incubated 15 min on after which 15μl of IP buffer (20mM Tris HCl pH 7.4, 2mM EDTA, 150mM NaCl, 0.1% Triton X-100) was added and sample was divided into 2 tubes, 10μl each for subsequent IP with Dynabeads bound with corresponding antibodies.

Two types of input samples were prepared. First, the samples were treated according to the RAT-ChIP protocol, but instead of immunoprecipitation, DNA was extracted using DNA Clean & Concentrator kit from Zymo Research, followed by tagging with Tn5 transposase according to manufacturer’s protocol (Illumina) and sequencing library construction using PCR. Second, samples were initially treated according to the RAT-ChIP protocol and tagging of chromatin was performed in cell nuclei right after treatment with restriction enzymes followed by DNA extraction and library generation using PCR as described above. Detailed RAT-ChIP protocol is available in S1 File and at protocols.io website (dx.doi.org/10.17504/protocols.io.69qhh5w).

### *In silico* analysis of restriction enzyme cutting sites

Human genome hg19 version GRCh37.p13 from EnsEMBL website (http://grch37.ensembl.org/) was used for the *in silico* analysis. The consensus sequence (‘AGCT’, ‘TTAA’, ‘CCWGG’—where ‘W* can be either ‘A’ or ‘T’, and ‘GANTC’—where ‘N’ can be any nucleotide) for each restriction enzyme (AluI, SaqAI, HinfI, MvaI) respectively, was mapped onto each chromosome sequence. The final list contained over 50 million restriction site positions. The recorded coordinates were used to determine the average number of restriction enzyme recognition sites per 1kb in chromHMM K562 chromatin state regions [23] (repeat containing states 14 and 15 were excluded from the analysis) using custom written Perl scripts followed by average fragment length calculation. Custom Perl scripts are available from https://github.com/reidar-andreson/nucleosomes. Coordinates for gap, repeat and chromHMM K562 chromatin state regions were downloaded from UCSC Table browser [24]. Qualimap [25] was used to get the quality metrics of different ChIP-seq datasets in S4 Table and for the calculation of median read length in K562 cell input sample in different chromatin state regions.

### Used publicly available data

Raw data from following K562 ChIP-seq datasets from GEO database were downloaded and reprocessed as described below: GSM945165, GSM945228, GSM733680, GSM733658, GSM1782695, GSM1782755, GSM1782693, GSM1782739, GSM1918612-GSM1918616, GSM1918602-GSM1918606, GSM1918592-GSM1918596, GSM1918582-GSM1918586, GSM1141671 and GSM1141672. bESC ChIP-seq data is form GSE110039 and K562 RNA-seq data from GSM1940168. For S13C Fig, bigwig files were downloaded from the following datasets—GSM2082690, GSM2082693, GSM2082696, GSM2082698, GSM2082701, GSM2082703. For ICM and TE gene expression comparisons gene lists from the following publications were used [26–30].

### RAT-ChIP-seq and expression data analysis

Sequencing reads were mapped to hg19 or bosTau8 genome using Bowtie2 (version 2.3.3.1) [31] using following parameters -k 2 -N 1. Next, bam files were sorted and indexed using SAMtools (version 1.6) [32] and bigwig files were created using deepTools 2.0 [33]. We used blacklist regions for hg19 genome annotation created during ENCODE project to exclude them from further analysis [34]. Bigwig files were visualized in UCSC genome browser as custom tracks [24]. Peak calling was done using SICER-rb.sh script (version 1.1) [35] with following parameters: (100 200 200 0.74 600 50). For the final list of peaks regions that overlapped with hg19 blacklisted regions were discarded. Differential peak calling was done using SICER-df-rb.sh script (version 1.1) [35] with following parameters: (100 200 200 0.74 600 50). In addition to the FDR threshold, arbitrary 4-fold cut-off was used for H3K27me3 signal to restrict the number regions for further analysis. Manipulation with genomic regions such as intersection or subtraction was done using bedtools (version 2.26.0) [36] or various operate on genomic intervals tools in the Cistrome Galaxy server [37,38]. Global pairwise Pearson correlation analysis, clustering and heatmap generation was performed in 5kb windows using multiple wiggle files correlation tool in Cistrome Galaxy server [37,38]. Global H3K4me3 heatmaps in 10kb regions around TSS (in 100bp windows) were generated using heatmap tool in Cistrome Galaxy server [37,38]. Average H3K4me3 enrichment profiles in 10kb regions around TSS (in 10bp windows) were generated using Sitepro tool in Cistrome Galaxy server [37,38]. Locus specific average metagene (3kb up and downstream of the gene and 6kb metagene body) enrichments of H3K27me3 were calculated and visualized using bwtool [39]. GO enrichment analysis of cell-type enriched histone modification regions (identified using SICER and sorted based on fold change) was performed using GREAT (version 3.0.0) [40] with proximal: 5 kb upstream, 1 kb downstream, plus distal: up to 100 kb, parameters. Venn diagrams were created using InteractiVenn [41]. For data visualization and statistical analysis MS Excel and GraphPad were used. Paired T-Test was used to calculate if average signals between pairs of corresponding gene regions (TE or ICM upregulated genes) in TE and ICM were significantly (p<0.05) different.

The datasets supporting the conclusions of this article are included within the article, its supplementary materials and in the Gene Expression Omnibus (GEO) repository, [GSE103734].

## Results

### Development of the RAT-ChIP method

**Fig 1:**
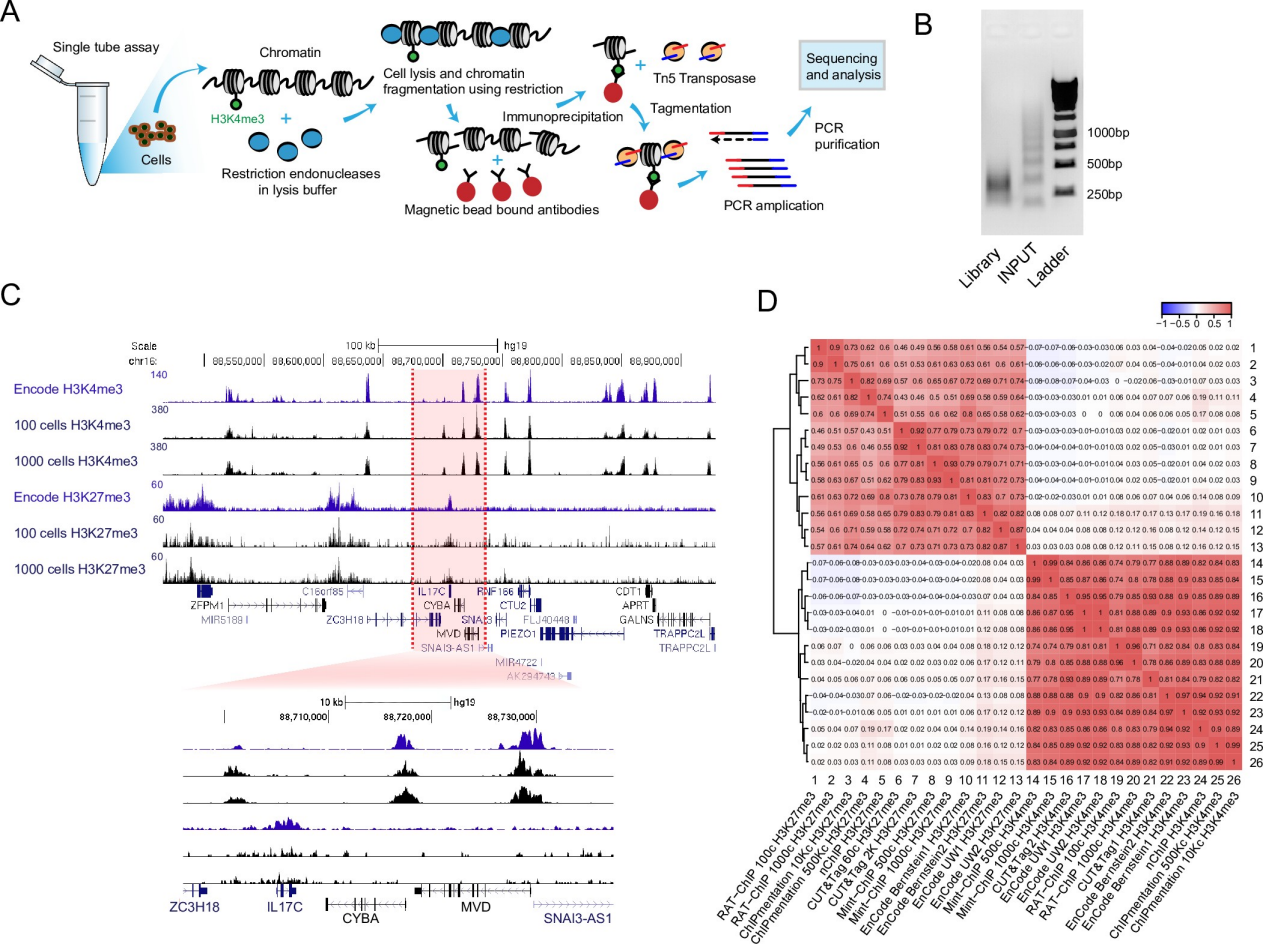
RAT-ChIP enables genome wide histone modification profiling from 100 cells. **A** Overview of RAT-ChIP method. **B** Agarose gel electrophoresis of DNA after chromatin treatment with a combination of restriction enzymes (middle lane) and after tagmentation (left lane). **C** UCSC genome browser custom histone H3K4me3 and H3K27me3 tracks of RAT-ChIP-seq with 100 and 1,000 K562 cells in comparison with ENCODE data in a genomic region centred around IL17C gene. **D** Clustered global Pearson correlation heatmap (enrichments in 5kb windows) of RAT-ChIP-seq and different published histone H3K4me3 and H3K27me3 datasets in K562 cells.

The outline of the RAT-ChIP method is depicted in Fig 1A. The whole protocol is designed to work as a single tube–one-day assay, reducing the number of necessary experimental steps, thus minimizing the loss of material. When designing the assay we aimed to use Tn5 transposase for sequencing library preparation as is used in ATAC-seq [42] and ChIPmentation method [43]. We therefore needed a method for chromatin fragmentation that would be compatible with both tagmentation and low cell numbers. Sonication is the most commonly used method chromatin fragmentation but it was not a good option in our case, as it cannot be done in small volumes using the standard equipment available in most labs. Larger volumes, in turn, lead to dilution of the material, which is undesirable when working with a small number of cells. Moreover, we wanted to avoid crosslinking, which due to harsh treatment needs to be done when using sonication, as it adds several additional steps to the protocol resulting in loss of material. We therefore turned to enzymatic digestion methods, which can be performed in considerably smaller volumes without the need for crosslinking (at least when working with histones). Micrococcal nuclease (MNase) digestion that is used in native ChIP protocols for chromatin fragmentation was in our case not optimal, as MNase digests the entire free DNA between nucleosomes resulting in inefficient adapter insertion when these nucleosomes will be used for tagging. Thus, we looked for alternative enzymatic means for chromatin digestion.

Restriction endonucleases have long been used for DNA footprinting to identify the locations of DNA bound proteins. They only cut at specific recognition sequences, preferably in between nucleosomes, and do not have exonuclease activity. Theoretically, even a single 4bp recognizing restriction enzyme should cut on average every 256bp but the actual cutting frequency depends on the DNA sequence and position of the nucleosomes. Nevertheless, a combination of frequently cutting restriction enzymes should allow achieving relatively even fragmentation coverage across the genome and fine enough resolution needed for histone modification profiling. We therefore tested an array of frequently cutting restriction endonucleases (S2 Table) for their ability to cut DNA in the chromatin context. All used enzymes were from Thermo Scientific FastDigest lineup, which allows for rapid 5-minute digestions. We used buffer conditions containing a low amount of non-ionic detergents that can disrupt cell and nuclear membranes but do not interfere with the enzymatic activity. Out of the 10 tested restriction endonucleases, 5 were able to cut chromatin with various efficiencies, as evidenced by the appearance of nucleosomal ladders on an agarose gel (S1A Fig). AluI and SaqAI were the most efficient cutters followed by MvaI, HinfI and BsuRI. Surprisingly, the other enzymes were not able to visibly cut the chromatin even if they were able to cut naked DNA under the same conditions (data not shown). Although the best cutting single enzymes relatively efficiently fragmented chromatin, a majority of the DNA was still too large for ChIP. We therefore cut chromatin using combinations of restriction enzymes and observed a decrease in average DNA size when more restriction endonucleases were used simultaneously (S1B Fig). Next, we tested if incubation time has an effect on fragmentation efficiency but did not see a substantial variation in DNA size between 5-, 10- and 15-minute incubation times (S1C Fig), indicating that 5 minutes is enough to digest majority of the DNA.

Since restriction enzymes recognize specific DNA sequences we used *in silico* analysis to identify the genome-wide cutting sites of the used restriction endonucleases based on hg19 genome build. Using a combination of 4 restriction endonucleases (AluI, SaqAI, MvaI and HinfI), 87% of the genome was predicted to be cut into smaller pieces than 1,000bp (S2A Fig). In total there were 2,465 regions that based on the *in silico* analysis remained larger than 1,000 bp. Out of these 273 overlapped with gap regions (regions with no annotated sequence in the hg19 genome build), 290 overlapped with blacklisted regions (regions that have abnormally high read counts in next-generation sequencing based studies) identified by ENCODE project and 2,065 overlapped with repeat regions downloaded from UCSC table browser RepeatMasker track (S3A Table). This left with only 299 regions that did not overlap with any of the three lists (S2B Fig and S3B Table), showing that at least *in silico* there are only a handful of unique genomic regions that cannot be effectively fragmented using a combination of restriction endonucleases.

We also compared *in silico* DNA fragment sizes that would be created by cutting with the 4 restriction enzymes with experimentally determined fragment sizes after cutting with the 4 restriction enzymes followed by tagmentation and sequencing. The analysis showed that regardless of the studied genomic regions with different chromatin states defined by chromHMM, the average/median size of DNA fragments was always smaller than 176bp–indicative of even and sufficient fragmentation for ChIP (S2C Fig).

Having identified that restriction enzymes could be used for chromatin digestion we proceeded with testing the RAT-ChIP protocol. The use of enzymatic digestion enabled us to considerably downscale the sample volume so that the digestion was performed in 1μl and immunoprecipitation (IP) was performed in 11μl final volume using 1μl of magnetic beads pre-bound with an antibody against the histone modification of interest. After IP, beads were washed and tagging was performed directly on the beads. After another round of washes, the beads were used directly in PCR reaction. Skipping the decrosslinking, proteinase K treatment and DNA purification steps minimizes the loss of DNA and allows working with very low amounts of material. Even when starting with only 100 cells, we easily obtained enough material for sequencing after 16 rounds of PCR (S3A Fig). Moreover, tagging on beads further decreases the fragment size, so that the bulk RAT-ChIP library was between 200 and 500bp in size (Fig 1B, S3B Fig), which is ideal for sequencing. Initial RAT-ChIP tests using 100 and 1,000 erythroleukemic K562 cells and H3K4me3 antibody followed by qPCR showed enrichment at the promoters of housekeeping genes *GAPDH* and *VSP29* compared to negative control regions (4^th^ exon of *ZNF7* gene and an intergenic region on chr17 (neg ctrl)) (S3C Fig) showing that using qPCR the method is capable of detecting histone modification enrichments from only 100 cells.

### RAT-ChIP enables high quality genome-wide histone modification profiling from 100 cells

We next coupled our RAT-ChIP protocol with Illumina sequencing for genome-wide histone modification profiling. We used human K562 cells derived from a chronic myelogenous leukemia for which many publicly available datasets exist allowing us to compare our method with others. We used 100 and 1,000 cells and antibodies that recognize H3K4me3 or H3K27me3 to see if RAT-ChIP can be used to study both active and inactive histone marks. After paired-end sequencing the reads were mapped to hg19 genome assembly, enrichment profiles were created and visualized as custom tracks in the UCSC genome browser. Visual inspection and comparison to the corresponding ENCODE data suggested that RAT-ChIP can produce high quality profiles that look similar to ENCODE data for both histone H3K4me3 and H3K7me3 modifications (Fig 1C).

To further assess the quality of the RAT-ChIP data we compared it to several other published ChIP-seq experiments that had data available with K562 cells. These included two datasets from ENCODE, a native ChIP-seq dataset (NCHIP), as well as three datasets from low input methods (see S4 Table) [15,34,43–46]. We downloaded raw sequencing data and processed all the datasets in the same way. Comparing various parameters such as % of mapped reads, GC% and fragment size for paired-end data showed that although there is variability between the compared datasets, RAT-ChIP performed comparably to other methods (S4 Table). One parameter that, as expected, is clearly dependent on the number of cells used is the percentage of duplicated reads. In here, the low input methods had more duplicated reads ranging from 30–64% (S4 Table).

Visualization in the UCSC genome browser showed that RAT-ChIP produces similar enrichment profiles with all the studied datasets (S4 Fig). To further assess how does RAT-ChIP compare to other methods we conducted clustering based on global correlations in 5kb windows between all the datasets. Two main clusters formed according to the studied histone H3 modification (Fig 1D). Overall, the following correlations were found between H3K4me3 (r2 = 0.71–1.00) and H3K27me3 datasets (r2 = 0.43–0.93). Within the modifications, unsurprisingly, datasets from the same lab showed higher correlations and usually clustered together. Global heatmaps of H3K4me3 signals around +- 5kb region of TSSs also showed similar profiles between RAT-ChIP and published datasets (S5A Fig).

To identify how RAT-ChIP signal intensities correlate with gene expression we divided genes into 3 groups based on RPKM values of published K562 cell-line RNA-seq data [47]. Average signal intensities around +- 5kb region of TSS correlated with gene expression status in all profiled datasets, expectedly, genes with higher expression had more H3K4me3 around their TSS. One striking difference that appeared with the average signal intensity profiles around TSS was that while majority datasets displayed a lower signal around TSS, known to harbour a nucleosome free-region, RAT-ChIP data, recently published CUT&Tag datasets [46] and to a lower extent MINT-ChIP [15] showed a clear signal in this region. This difference might be caused by both biological and technical reasons. The signal at the TSS in RAT-ChIP data could be reduced if fragments in between 120–420 bp were analyzed or if input sample signal was subtracted from the IP signal but not when subtracting H3 RAT-ChIP signal (S5B Fig).

Average H3K27me3 levels in metagene plots, opposite to H3K4me3 data, negatively correlated with gene expression—in all datasets, the promoter and whole gene body had higher levels of H3K27me3 signal in genes with lower expression (S6 Fig). However, the shape of the average profiles varied from dataset to dataset. With RAT-ChIP data, the average signal was very little influenced by exclusion of small (<120) and large (>420) fragments but was markedly influenced by subtracting H3 signal and clearly over compensated when subtracting input signal (S6 Fig). These data collectively show that RAT-ChIP produces comparable H3K4me3 and H3K27me3 profiles to published data, and manages to capture the known properties of these two histone marks.

To assess the reproducibility of the method we created additional 3 replicates using 100 and 1000 K562 cells and H3K4me3 and H3K27me3 antibodies (S7 Fig). Pairwise comparison of the replicates ChIP signal in 5kb windows showed that, expectedly, there is more variation between samples with 100 cells (Pearson correlation coefficients 0.69–0.81 for H3K4me3 samples and 0.67–0.76 for H3K27me3 samples) compared to 1000 cells (Pearson correlation coefficients between 0.93–0.97 for H3K4me3 samples and 0.84–0.95 for H3K27me3 samples) (S8 Fig).

We next determined regions enriched for histone H3K4me3 using SICER and overlapped the regions between different samples (S5 Table). When one of the ENCODE datasets (UW1) was used as a reference RAT-ChIP H3K4me3 peaks overlapped with 72–73% of the reference peaks which was in the same range with Mint-ChIP (68–73%), CUT&Tag (66–74%) and NChIP (71%) but lower than with other methods with more cells (82–90%) and a replicate from the same lab (93%) (S9A Fig). This analysis shows that despite of lower overlap, low input methods are still capable of identifying majority of the enriched regions. Moreover, the regions not identified, have on average much lower signal in the original ENCODE data, suggesting that RAT-ChIP misses regions with low H3K4me3 enrichment (S9B Fig).

### RAT-ChIP can identify differences in histone modification profiles between cell-lines

**Fig 2:**
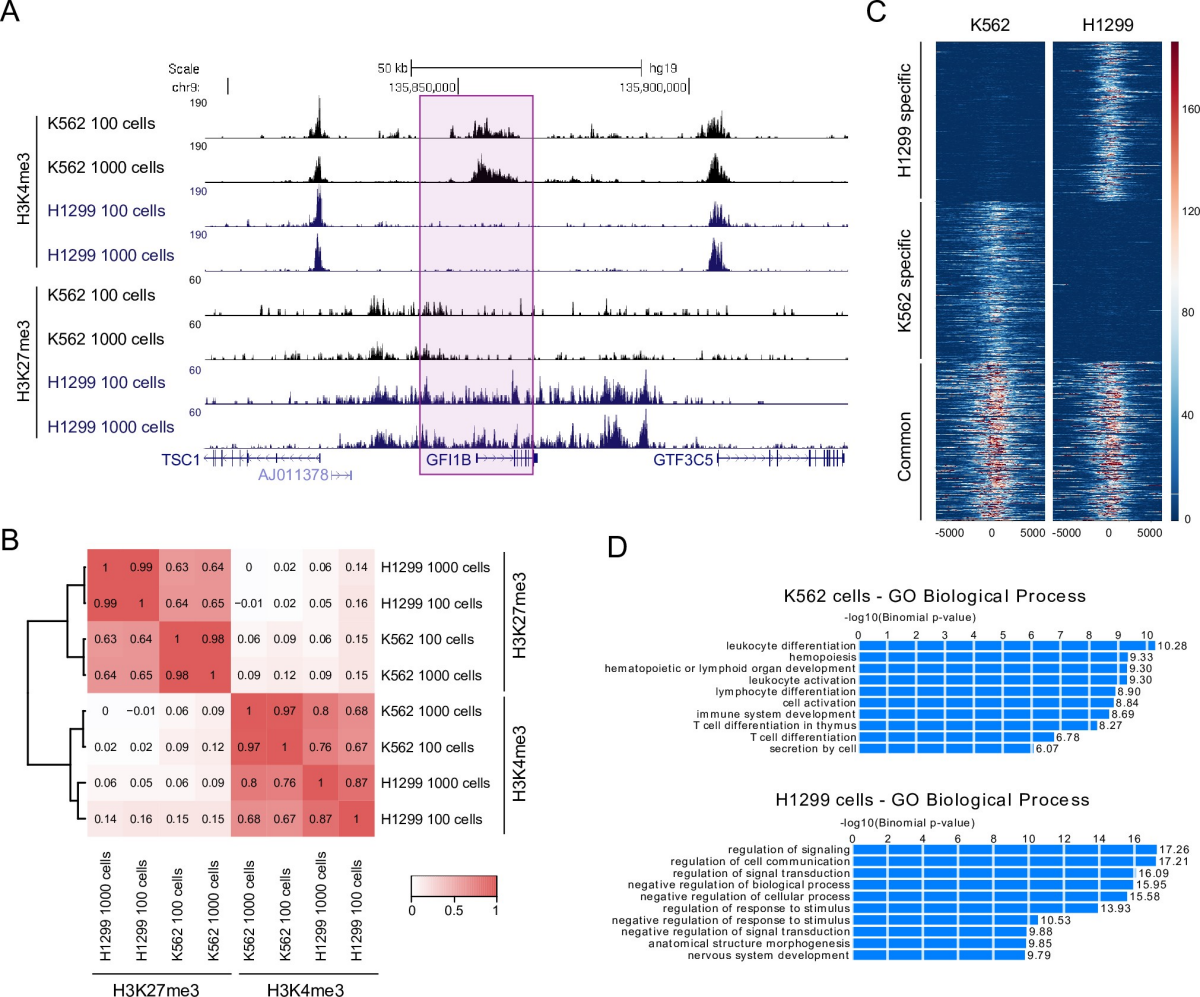
RAT-ChIP can identify differences in histone modifications between cell-lines. **A** UCSC genome browser custom histone H3K4me3 and H3K27me3 tracks of RAT-ChIP-seq with 100 and 1,000 cells in K562 and H1299 cells. **B** Clustered global Pearson correlation heatmap of histone H3K4me3 and H3K27me3 datasets of K562 and H1299 cells. **C** Heatmap of histone H3K4me3 signal in K562 and H1299 cells in 4kb region centered around the TSS of 300 genes with either cell type specific or common signal. **D** Enriched terms of GREAT GO analysis of top 500 peaks differentially enriched between K562 and H1299 cells.

Having identified that RAT-ChIP-seq data from K562 cells are comparable to other published datasets we next studied if it is capable of identifying cell type specific differences in histone modification profiles. To this end we performed RAT-ChIP-seq with 100 and 1,000 cells in human non-small cell lung carcinoma cell line H1299, for which no published ChIP-seq data exist, using histone H3K4me3 and H3K27me3 antibodies. After alignment and filtering, bigwig files were created and visualized in UCSC genome browser. Visual inspection and comparison of the RAT-ChIP tracks from H1299 and K562 cells showed similar enrichment profiles at the transcriptional start sites (TSS) of genes for H3K4me3 and broad H3K27me3 domains (S10A Fig). Inspecting the loci of known hematopoietic transcription factors such as *GFI1b* (Fig 2A), *GATA1* (S10B Fig), *LMO2* (S10C Fig), *ETO2* (data not shown) and entire globin locus (S11A Fig), revealed clear differences between the two cell lines. For example, in *GFI1b* locus there was enrichment of H3K4me3 around the TSS in K562 cells but the modification was completely absent in H1299 cells (Fig 2A). The opposite was seen with H3K27me3, where a region around *GFI1b* gene had clearly higher signal of H3K27me3 in H1299 cells compared to K562 cells (Fig 2A). Similar examples could be found for H1299 cell-line—several genes involved in epithelial to mesenchymal transition (EMT) such as *TWIST2* and *SIX1*, showed elevated histone modification profile of active genes in H1299 cells (S11B and S11C Fig). Pairwise correlation and clustering analysis showed that samples clustered first according to the profiled histone modifications and within the modifications according to cell-lines (Fig 2B). To gain a more global view of the differences we identified differential H3K4me3 peaks between the two cell lines. Fig 2C shows heatmap of the signal intensities around TSS of 300 genes, which were differentially modified in one of the cell lines in contrast with 300 random genes that were not differentially modified. To see if the differentially modified regions are near functionally relevant genes we performed a GO enrichment analysis using GREAT [40]. Analysis of 500 top H3K4me3 peaks that were more enriched in one of the cell lines compared to the other revealed enrichment of hematopoiesis related terms for K562 cells and signalling related terms for H1299 cells in biological processes category (Fig 2D). Similar analysis was performed with H3K27me3 mark (S12A and S12B Fig). Regions with higher signal (2840 regions) in K562 cell line were enriched in genes associated with cellular movement associated GO terms and regions with higher signal in H1299 cells (2350 regions) were enriched in hematopoiesis related GO terms. This analysis shows that RAT-ChIP-seq can identify hundreds of tissue specific genes with different histone modification profiles between K562 and H1299 cells.

### RAT-ChIP enables histone profiling of blastocyst stage bovine embryos

Recently, our group has used bovine as a model to study the molecular events that take place during of early embryogenesis of large mammals–chromosomal instability in particular [21]. We therefore aimed to put RAT-ChIP-seq to test and profile histone H3K4me3 and H3K27me3 modifications in blastocyst stage embryos. Thus far, mouse is the only mammal, which embryos have been used for genome-wide histone profiling at such an early stage of development [16,17,20]. Using *in vitro* fertilized embryos, we used micromanipulator in combination with laser microdissection to separate blastocysts into inner cell mass (ICM) and trophectoderm (TE) fractions. Pooled material of three embryos was subsequently used for RAT-ChIP-seq experiments. After alignment to bosTau8 genome, bigwig tracks with enrichment profiles were created and visualized in UCSC genome browser next to recently published histone modification data from bovine embryonic stem cells (bESCs) [48]. As expected, histone H3K4me3 was enriched mostly around promoter regions while histone H3K27me3 had broad domains of enrichment as exemplified by looking at the locus centred around housekeeping gene *GAPDH* (S13A Fig), showing that RAT-ChIP can be used to obtain genome-wide histone modification profiles from early developmental stage embryos.

**Fig 3:**
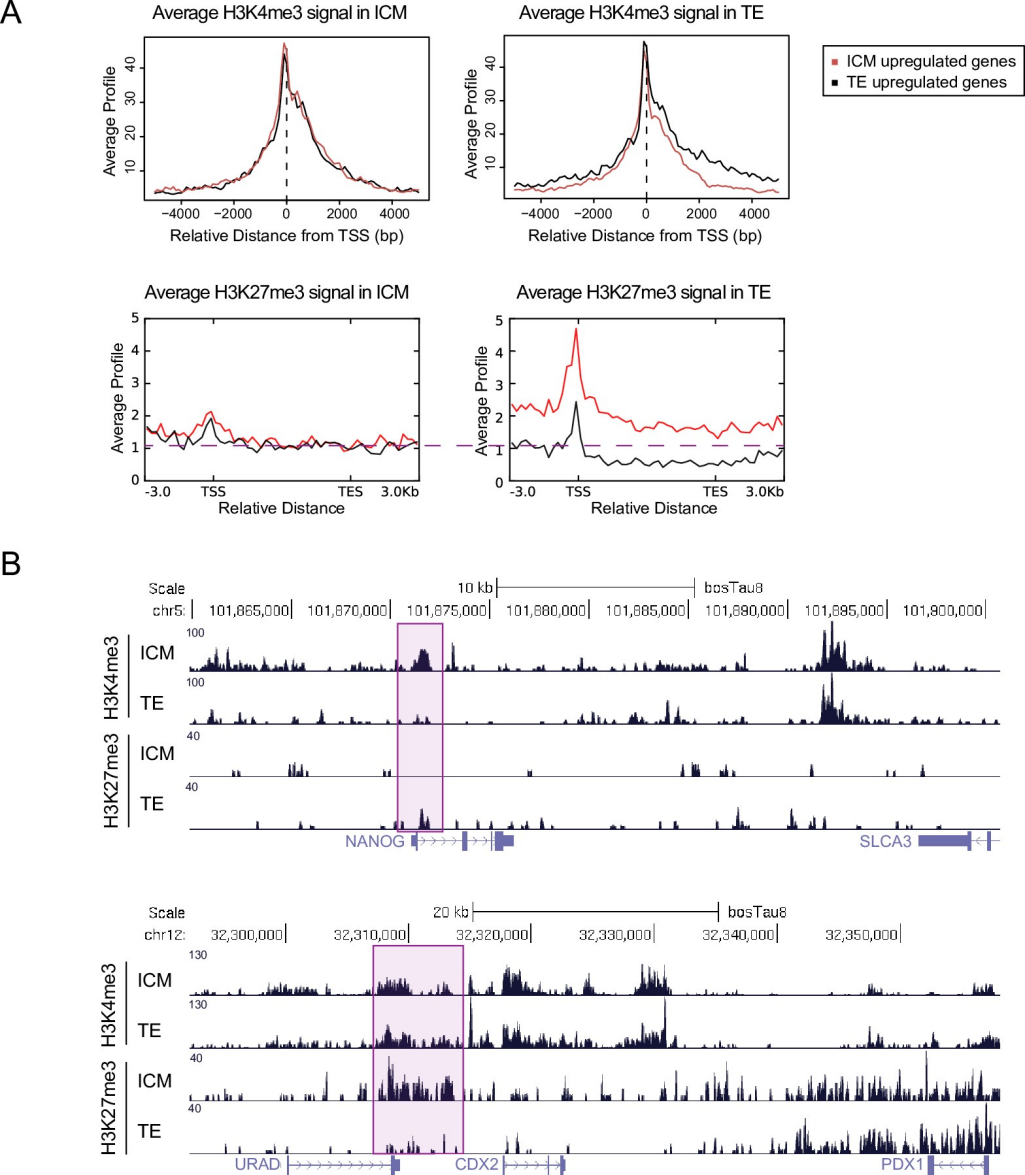
Histone H3K4me3 and H3K27me3 modification profiles of ICM and TE of blastocyst stage bovine embryos. **A** Average histone H3K4me3 (upper panels) and H3K27me3 (lower panels) profiles around TSS of genes that are upregulated in ICM (red line) or TE (black line) in ICM (panels on the left) and TE (panels on the right). **B** UCSC genome browser custom histone H3K4me3 and H3K27me3 tracks of bovine blastocyst ICM and TE in *NANOG* (upper) and *CDX2* (lower) gene regions.

To gain more global view of how these two histone marks act in regulation of gene expression we intersected our histone modification data with published gene expression data from ICM and TE of bovine blastocysts. We found five studies where gene expression profiles of ICM and TE were compared [26–30]. Two of them used RNA–seq and two others Affymetrix microarrays. In addition, one of the studies compared *in vivo* and *in vitro* derived blastocysts, making it in total six datasets. We obtained the published lists of differentially expressed genes between ICM and TE for all the studies and intersected them. Overall, the overlap was relatively modest–there were only 6 and 0 gene(s) that were consistently upregulated in ICM and TE, respectively, in all 6 datasets. The same numbers for at least 5 overlapping datasets were 28 and 5 and for at least 3 overlapping datasets 210 and 221 for ICM and TE, respectively (S13B Fig, S6 Table). This analysis shows that there is a lot of variability and that the changes between TE and ICM at the transcriptome level are not huge at this early stage. In order to link gene expression to histone modification profiles we took the genes that were differentially expressed between ICM and TE at least in 3 datasets and profiled the average histone H3K4me3 profiles around 5kb regions around TSS and created metagene plots for H3K27me3 encompassing gene body and 3kb up and downstream of TSS and TES, respectively (Fig 3A). In ICM, both groups of genes had similar levels of H3K4me3 and H3K27me3. In trophectoderm, TE upregulated genes had higher average levels of H3K4me3 around their promoters and lower levels of H3K27me3 levels around the whole gene region compared to genes upregulated in ICM (Fig 3A), suggesting for a more pronounced epigenetic regulation. The differences seen between ICM and TE are not due to major differences in immunoprecipitation quality as both H3K4me3 and H3K27me3 average signal was clearly associated with gene expression levels in both datasets (S14A Fig).

The involvement of epigenetic regulation in ICM and TE specific gene expression is further supported by the analysis of average signals between pairs of corresponding gene regions (TE or ICM upregulated genes) in TE and ICM. In all cases there were statistically significant differences—ICM upregulated genes had more H3K4me3 and less H3K27me3 signal in ICM compared to TE and, *vice versa*, TE upregulated genes had more H3K4me3 and less H3K27me3 signal in TE compared to ICM (S14B Fig).

Although between samples the changes in histone modifications are in the expected direction, the changes are relatively modest–on a single gene level SICER managed to identify higher H3K4me3 on the TSS of 77 genes out of 210 (37%) ICM upregulated genes in ICM and on the TSS of 82 genes out of 221 (37%) for TE upregulated genes in TE. The same numbers for TSS where H3K4me3 levels were upregulated at least 2x were 17 (8%) and 12 (5.4%) for ICM and TE upregulated genes respectively (S6C and S6D Table). To identify genes, which are potentially polycomb regulated, we calculated average H3K27me3 levels for ICM and TE upregulated genes for regions spanning the whole gene plus 2kb upstream and for a region +-2kb of TSS. We identified 6 (gene+2kb upstream) and 17 (+-2kb of TSS) genes where the changes in H3K27me 3 levels were at least 4 times higher in TE compared to ICM for ICM upregulated genes and 23 (gene +2kb upstream) and 24 (+-2kb of TSS) genes where the changes in H3K27me 3 levels were at least 4 times higher in ICM compared to TE for TE upregulated genes (S6C and S6D Table).

To understand how the data can be used to learn about the epigenetic regulation of individual genes, we focused on genes known to be important in either ICM or TE specification and function. The promoter region of *NANOG*, a well-known pluripotency gene in embryonic stem (ES) cells was in our combined list of ICM upregulated genes and had a H3K4me3 peak in ICM but not in TE (Fig 3B). This is different from a recently published data from mouse where *Nanog* promoter region is enriched for H3K4me3 in both ICM and TE (S15A Fig) [20]. Another good example where we detected difference in H3K4me3 levels around the promoter region is *DPPA3*, although it was differentially expressed between TE and ICM in only one of the five published transcriptome studies (S15B Fig, S6B Table). In contrast, enrichment of H3K4me3 seen in ICM sample was absent from the promoter of *DPPA3* gene in a recently published data with bovine embryonic stem cells (S15B Fig) [48] and was present in both ICM and TE of mouse blastocysts (S15C Fig).

Similar to *DPPA3*, the master regulator of TE development, *CDX2* [49], was in the list of TE upregulated genes in only one of the expression datasets (S6B Table) and was enriched for H3K4me3 in both cell types. Interestingly, there was an enrichment of H3K27me3 upstream of *CDX2* gene specifically in ICM. The same region came up as the first hit when BLAT alignment was performed using a sequence of the recently characterized mouse trophectoderm specific enhancer upstream of CDX2 gene [50], suggesting that *CDX2* might be down regulated in ICM through Polycomb-mediated enhancer repression. These examples show that RAT-ChIP-seq data can be used for hypotheses generation to identify molecular mechanisms driving gene regulation in early embryonic development.

## Discussion

We have developed a novel low input ChIP method called RAT-ChIP that can be used to create genome-wide histone modification profiles from only 100 cells. There are several important modifications to the standard protocol that enabled us to achieve successful results with such a low number of cells.

First, the use of restriction enzymes for chromatin fragmentation enabled us to keep the sample volumes small, which is essential when working with low amount of starting material. The volumes used in other published protocols, except for these that use dedicated equipment [13] not readily available, use much higher volumes. In addition, due to small volumes the reagent costs are reduced significantly. For example, in a typical ChIP experiment 30μl of magnetic beads are used compared to 1μl in RAT-ChIP. Similar to MNase, restriction enzymes only cut in between nucleosomes but in contrast to MNase they leave DNA overhangs that can be used for sequencing library generation by tagmentation using chromatin as a template. Restriction endonucleases have been used in DNA fingerprinting studies for years. More recently they have been successfully used in different chromosome conformation capture (3C) based methods to assess the 3D chromatin architecture [51]. The drawback of using restriction enzymes is that the cutting is not random. However, combining several frequently cutting enzymes enables to optimize the coverage and desired fragment size. As restriction endonucleases are sequence-specific and only cut in between nucleosomes there is no problem of over digestion. Moreover, due to the sequence specificity it is possible to predict genome-wide cutting sites. In combination of the four restriction endonucleases used in this study based on *in silico* analysis most of the genome is fragmented to the size suitable for ChIP. The larger fragments that remain often overlap with gaps, ENCODE project identified black regions (regions that have abnormally high number of reads in next generation sequencing data) or repeats that are difficult to analyze. Moreover, the larger regions that can cause false positive signals can be identified *in silico* and removed from further analysis. A recently published method called RELACS also used a similar approach to us showing that restriction endonucleases can be used for chromatin fragmentation in ChIP assays [52].

Second–minimization of steps where material could be lost, such as centrifugations and DNA extractions. This was achieved by omitting several steps in regular ChIP protocols such as crosslinking and proteinase K treatment. All steps in a protocol are carried out in a single tube so that the first DNA purification occurs only after PCR, when loss of material is not anymore an issue.

Third—simple, one step library preparation. We adapted the first step of library generation step from the ChIPmentation method. In addition, being extremely simple and cost effective, due to the random nature of tagmentation it allows to further decrease DNA size, so that majority of the fragments in the final library come from single nucleosomes. In contrast to ChIPmentation, we performed PCR directly on magnetic beads using the bound chromatin as a template, similar to recently published high-throughput ChIPmentation [53] and lobChIP [54]. Skipping DNA purification avoids loss of material. The method is also very fast taking less than a day to complete. Recently, several methods–CUT&RUN, CUT&Tag, ChIL–seq, scChIC-seq and CoBATCH have been published that look really promising, as they have been shown to be able to obtain data from single cells [46,55–59].

Using K562 cells, we showed that the histone H3K4me3 and H3K27me3 profiles created using RAT-ChIP compare well to other published datasets, demonstrating that it can be used to profile chromatin marks associated with both active and inactive genes. One difference we observed in RAT-ChIP H3K4me3 data compared to regular ChIP datasets was a prominent signal exactly at TSS. Even higher signal in this region was also seen in CUT&Tag H3K4me3 data, but not in ChIPmentation data both of which use Tn5 transposase. To a lesser extent, the signal was also present in H3K4me3 Mint-ChIP data. All the three methods that display the peak do not use crosslinking and use mild conditions for chromatin fragmentation. In case of expressed genes, the region surrounding TSS is more accessible and usually considered to be depleted of nucleosomes [60]. Therefore, part of the signal definitely comes from higher accessibility. We do see a signal in this region if we prepare input samples by tagging restriction enzyme digested chromatin within cell nuclei. However, the input prepared this way resembles ATAC-seq and is not ideal control as in case of RAT-ChIP the tagging takes place on beads after chromatin precipitation. The fact that the signal is still present after exclusion of small fragments (<120bp) suggests that this region might contain histone proteins that are not detectable with regular ChIP assays. Indeed, several papers have reported for the presence of MNase sensitive -1 nucleosome [61–64]. Our data agree with these reports and suggests that this nucleosome is H3K4me3 methylated.

One issue that arises with ChIP methods that use on-bead tagmentation is what kind of control samples to use, as input cannot be treated the same manner as immunoprecipitation samples. In our case, tagging input samples in intact nuclei right after restriction enzyme treatment creates a profile that resembles ATAC-seq data and tagging after DNA extraction misses the accessibility created bias. Tagmentation method, has opted for the use of IgG controls, however, IgG samples are not considered the best controls for sequencing based approaches [65]. Because of the low amount of starting material we failed to obtain good quality libraries with IgG samples. Another option is to use immunoprecipitation with H3 antibody as a control since these samples are treated identical to the samples of interest and give more even coverage compared to IgG. It has been shown that using INPUT or H3 pull-down as a reference has small differences with a negligible impact on the quality of a standard analysis [66]. Moreover, several reports have shown that using an input has a little if any advantage over not using an input [67–70]. Some studies imply that they do even worse and should be used only for peak prioritization [71]. The benefits or caveats of using input are probably case specific. For example, for pairwise comparison of samples to find regions with differential enrichments, input is not crucial, as biases present in both samples should cancel each other out. More care should be taken when quantitative statements are being made about the amounts of modifications. In this case even proper input might not be sufficient and using spike-ins should be considered if global changes in histone modification patterns are excepted [72,73]. Considering the above, we recommend using immunoprecipitation with H3 as the first choice for a control sample. Moreover, analysis should be done with and without using a control and attention should be paid when the regions of interest show differences depending on the analysis. In this case, an alternative method should be used to verify the results.

Replication experiments showed that RAT-ChIP can constantly obtain data from as few as 100 cells, however there is more experimental variation with such a low number of cells and thus replicates are especially important to make biologically valid conclusions.

By profiling histone H3K4me3 and H3K27me3 modifications in H1299 cells and comparing it to the data from K562 cells we showed that RAT-ChIP can identify epigenetic differences between cell lines.

We also showed that RAT-ChIP works on small numbers of primary cells by applying it to blastocyst stage bovine embryos. In addition to the vast potential bovine has in farming and biomedicine, it also serves as a good model system to study the molecular events that take place during early embryogenesis as its development is more similar to human compared to other common model organisms such as mouse [74]. Majority of the epigenetics experiments, genome-wide studies in particular, have been performed in mouse. Using RAT-ChIP we have created the first genome wide histone H3K4me3 and H3K27me3 modification profiles of ICM and TE of blastocyst stage bovine embryos. Considering that day 7–8 embryos consist on average about 125 cells (80 in TE and 45 in ICM) [45,46] and we used material from 3 pooled embryos for 2 ChIPs, only 70–120 cells were used per one immunoprecipitation.

Combined analysis of our histone modification data with lists of ICM and TE upregulated genes from published papers showed that gene expression changes are on average reflected by expected changes in histone modifications. The average differences in H3K4me3 levels are more pronounced in regions adjacent to TSS, especially downstream. This is in agreement with several recent reports that have associated breadth of H3K4me3 domains with cell identity and transcriptional activity [20,75]. Due to several reasons, however, such as low levels of enrichment in some regions, not completely pure cell populations, cellular heterogeneity and biological similarity, the changes at epigenetic level are not huge between ICM and TE. Nevertheless, looking at the histone modification profiles of factors with known importance either in ICM or TE function we could see evidence for the involvement of epigenetics in gene regulation. For example, a well-known pluripotency factor NANOG TSS had H3K4me3 only in ICM but not in TE. Similarly, it was shown recently that there is loss of H3K4me3 on *NANOG* gene upon human embryonal carcinoma NT2/D1 cell differentiation towards neural progenitors [76]. This is different from mouse blastocysts where the whole *Nanog* gene is covered with H3K4me3 both in ICM and TE (S15A Fig) [20]. Similarly, there were no differences in H3K4me3 signal at the promoter of *DPP3A* gene between ICM and TE in the mouse data, while our bovine data showed a signal only in ICM. This is again different from a recently published bES cell data where the promoter region of *DPP3A* was devoid of H3K4me3. *DPP3A* has a known conserved function of protecting the female genome from TET3 activity, it has role in pluripotency maintenance and reprogramming [77] and has been shown to be essential for bovine embryonic development [78]. The observed differences might be explained by locus specificity, timing, cell-type, cell purity used in ChIP experiments, or interspecies differences. Indeed, it was shown recently that there are interspecies differences upon ablation of *OCT4* gene between mouse and human, including the regulation of *NANOG* gene expression [79]. There are also several examples of differentially regulated genes between ICM and ES cells [80]. These examples show that there are important cell type and interspecies differences that need to be taken into account when drawing conclusions about the regulation of specific genes.

At the moment we have tested RAT-ChIP only with histone modifications. It remains to be studied if it could be also used to profile other chromatin bound proteins, including transcription factors. As the interaction of transcription factors is in general more labile, crosslinking step is usually used in ChIP. However, there is a recent protocol called ORGANIC ChIP, which demonstrated that transcription factors can be also immunoprecipitated without the need for crosslinking [81]. Moreover, recent CUT&RUN and CUT&Tag methods do not use crosslinking and work with transcription factors [46,55,56].

With the publication of several novel ultra-low input ChIP methods it is important to note that each method has its own characteristics, using small amount of material inevitably creates more variability and thus direct comparison of results obtained with different methods can be complicated. For example, Tn5 transposase based methods can be influenced by chromatin accessibility and correction for this bias with input is not always straightforward. Therefore, to confirm biological significance, replicate experiments are crucial and results obtained with one method should be repeatable by a different method.

In summary, we have developed a novel simple yet sensitive RAT-ChIP method that it can be used to study genome-wide histone modifications from less than 100 cells. Using the new method we have created the first genome wide histone H3K4me3 and H3K27me3 profiles in blastocyst stage bovine embryos that serve as a resource for further studies.

## Supporting information

S1 Fig Restriction enzymes can be used for chromatin fragmentation. **A** Restriction of chromatin using 10 frequently cutting restriction endonucleases. **B** Combining more restriction endonucleases in a single reaction results in more efficient chromatin fragmentation. **C** Test of restriction enzyme 5-, 10- and 15-minute incubation times shows that 5 minutes is sufficient to fragment majority of the chromatin.
(PDF)

S2 Fig *In silico* analysis of fragment sizes produced by 4 restriction enzymes in hg19 genome assembly. **A** Predicted fragment size distribution following cutting with AluI, SaqAI, MvaI and HinfI restriction endonucleases. **B** Size distribution of 299 fragments that remain larger than 1,000bp after *in silico* cutting with AluI, SaqAI, MvaI and HinfI restriction endonucleases. **C** *In silico* and experimental distribution of DNA fragment sizes in genomic regions with different chromatin states defined by chromHMM.
(PDF)

S3 Fig RAT-ChIP can identify histone H3K4me3 modification enrichments from 100 cells. **A** Average yield of RAT-ChIP libraries from 100 and 1,000 cells after 16 rounds of PCR. **B** Example RAT-ChIP library analysed using TapeStation. **C** RAT-ChIP enrichments of H3K4me3 at the promoters of *GAPDH*, *VPS29* and *ZNF7* genes compared to negative control region using 100 and 1,000 cells. A representative experiment is shown.
(PDF)

S4 Fig RAT-ChIP enrichment profiles compared to other publicly available datasets. Custom UCSC tracks of histone H3K4me3 and H3K27me3 profiles in *ETO2* (*CBFA2T3*) gene locus in K562 cells to compared RAT-ChIP with other publicly available datasets.
(PDF)

S5 Fig RAT-ChIP enrichment profiles compared to other publicly available datasets. **A** Heatmaps of H3K4me3 signal in RAT-ChIP and published datasets in 10kb genomic regions around TSS of all NCBI RefSeq genes ranked by H3K4me3 signal intensity in RAT-ChIP 100 cell sample. **B** Average H3K4me3 signal intensities around 10kb region of NCBI RefSeq genes divided into 3 equally sized groups (high, medium and low expression) based on their expression levels using published RNA-seq experiment [47] RPKM values. RAT-ChIP data was additionally processed by subtracting either H3 or INPUT signal from the H3K4me3 signal or restricting analysis to reads with fragment sizes between 120 and 420 bp.
(PDF)

S6 Fig RAT-ChIP enrichment profiles compared to other publicly available datasets. Average H3K27me3 signal intensities around 6kb metagene gene body and 3kb up and downstream of TSS (transcription start site) and TES (transcription end site) regions of NCBI RefSeq genes divided into 3 equally sized groups based on their expression levels (high, medium and low expression) using published RNA-seq experiment [47] RPKM values. RAT-ChIP data was additionally processed by subtracting either H3 or INPUT signal from the H3K4me3 signal or restricting analysis to reads with fragment sizes between 120 and 420 bp.
(PDF)

S7 Fig RAT-ChIP enrichment profiles of 4 replicate experiments. Custom UCSC tracks of histone H3K4me3 and H3K27me3 profiles depicting 4 replicate RAT-ChIP experiments with 100 and 1000 cells in a genomic region surrounding *GAPDH* gene.
(PDF)

S8 Fig Correlation analysis of replicate RAT-ChIP experiments. Scatterplots of pairwise comparisons of 8 replicate experiments (4 with 100 cells and 4 with 1000 cells) of histone H3K4me3 (**A**) and H3K27me3 (**B**) genome-wide signals in 5kb windows and corresponding Pearson correlation coefficients are shown.
(PDF)

S9 Fig RAT-ChIP H3K4me3 peak comparison with published datasets. **A** Percentage of overlapping H3K4me3 SICER peaks of RAT-ChIP and published datasets using ENCODE UW1 or Bern1 peaks as a reference. **B** Average H3K4me3 profiles in UW1 dataset around peaks that overlap (red line) or do not overlap (black line) with RAT-ChIP data show that RAT-ChIP missed ENCODE peaks are low in enrichment.
(PDF)

S10 Fig RAT-ChIP can identify cell type specific histone profile differences. Custom UCSC tracks of histone H3K4me3 and H3K27me3 profiles in *VPS29* (**A**), *GATA1* (**B**) and *LMO2* (**C**) gene loci in K562 cells compared to H1299 cells.
(PDF)

S11 Fig RAT-ChIP can identify cell type specific histone profile differences. Custom UCSC tracks of histone H3K4me3 and H3K27me3 profiles in hemoglobin (**A**), *TWIST2* (**B**) and *SIX1* (**C**) gene loci in K562 cells compared to H1299 cells.
(PDF)

S12 Fig RAT-ChIP can identify differences in histone modifications between cell-lines. **A** Heatmap of histone H3K27me3 signal in K562 and H1299 cells in 10kb regions centered around TSS of 300 genes with either cell type specific or common signal. **B** Enriched biological processes GO terms of GREAT analysis of differentially enriched regions between K562 and H1299 cells.
(PDF)

S13 Fig RAT-ChIP can identify histone H3K4me3 and H3K27me3 modification profiles from bovine blastocysts. **A** Custom UCSC tracks of histone H3K4me3 and H3K27me3 RAT-ChIP profiles in *GAPDH* gene locus in ICM and TE of blastocyst stage embryos compared to published bESC data. **B** 6-way Venn diagram to show overlaps of genes from six published datasets that are upregulated in bovine blastocyst stage ICM (left) or TE (right). Below the Venn diagram is a summary of number of genes that overlap with a shown number of experiments.
(PDF)

S14 Fig RAT-ChIP H3K4me3 and H3K27me3 enrichment profiles in bovine ICM and TE correlate with gene expression. **A** Average H3K4me3 (10kb around TSS) and H3K27me3 (6kb metagene body and 3kb up and downstream of TSS and TES, respectively) signal in NCBI RefSeq gene regions divided into 3 equally sized groups (high, medium and low expression) based on their expression levels using published RNA-seq experiment [47] RPKM values in ICM or TE. Plots are shown for ICM, TE and published bESC data [48]. **B** Scatterplots of H3K4me3 (4kb region surrounding TSS) and H3K27me3 (gene body and 2kb upstream of TSS) average signal with mean and SD are shown for TE and ICM upregulated genes. Paired T-Test was used to calculate if average signals between pairs of corresponding gene regions (TE or ICM upregulated genes) in TE and ICM are significantly different. * p<0.05, **** p<0.0001
(PDF)

S15 Fig RAT-ChIP data from bovine ICM and TE enables identification of cell type and species-specific differences in histone modifications. **A** Custom UCSC tracks of histone H3K4me3 and H3K27me3 profiles in *Nanog* gene locus in ICM and TE and morula of blastocyst stage mouse embryos [20]. **B** Custom UCSC tracks of histone H3K4me3 and H3K27me3 RAT-ChIP profiles in *DPPA3* gene locus in ICM and TE of blastocyst stage embryos compared to published bESC data [48]. **C** Custom UCSC tracks of histone H3K4me3 and H3K27me3 profiles in *Dppa3* gene locus in ICM and TE and morula of blastocyst stage mouse embryos [20].
(PDF)

S1 File RAT-ChIP-seq protocol. (PDF)

S1 Table List of selected low input ChIP-seq methods. (XLSX)

S2 Table List of tested Thermo Scientific FastDigest restriction endonucleases. (XLSX)

S3 Table List of regions larger than 1kb after *in silico* restriction. **S3A.** List of regions that remain larger than 1kb after *in silico* restriction with AluI, SaqAI, MvaI and HinfI restrictases. **S3B.** Additional information about 299 of regions that remain larger than 1kb after *in silico* restriction with AluI, SaqAI, MvaI and HinfI restrictases and do not overlap with gaps, repeat or blacklisted regions.
(XLSX)

S4 Table Statistics and quality parameters of RAT-ChIP-seq and reanalyzed ChIP-seq datasets in K562 cells. (XLSX)

S5 Table Number and overlap of H3K4me3 peaks of RAT-ChIP-seq and reanalyzed ChIP-seq datasets in K562 cells. (XLSX)

S6 Table Integration of RAT-ChIP and published expression data of bovine ICM and TE. **S6A.** List of genes upregulated in ICM compared to TE. **S6B.** List of genes upregulated in TE compared to ICM. **S6C.** List of genes and their TSS coordinates that are upregulated in ICM compared to TE in at least 3 different datasets. **S6D.** List of genes and their TSS coordinates that are upregulated in TE compared to ICM in at least 3 different datasets.
(XLSX)
