# Supplementary figures and images for: Genome-wide histone modification profiling of inner cell mass and trophectoderm of bovine blastocysts by RAT-ChIP
Source: PLoS One. 2019 Nov 25;14(11):e0225801. doi: 10.1371/journal.pone.0225801 (PMC6876874; doi:10.1371/journal.pone.0225801)

A

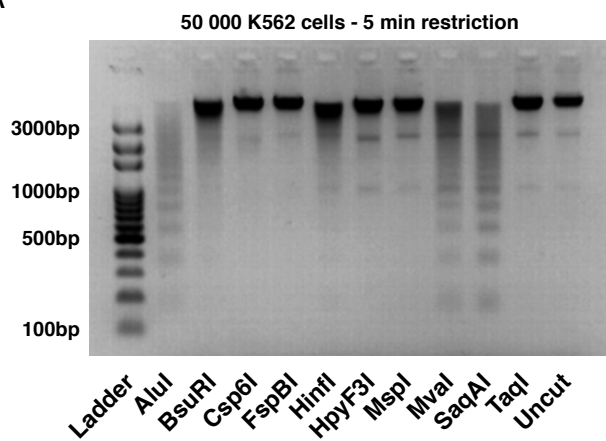

B

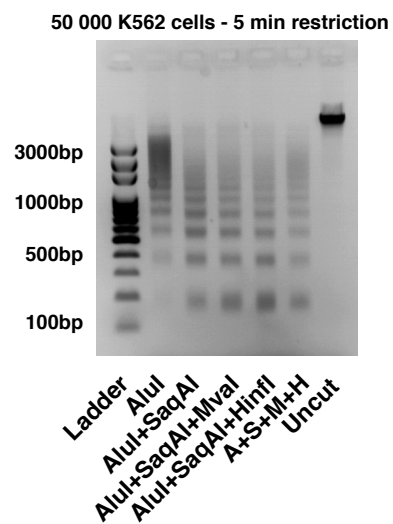

C

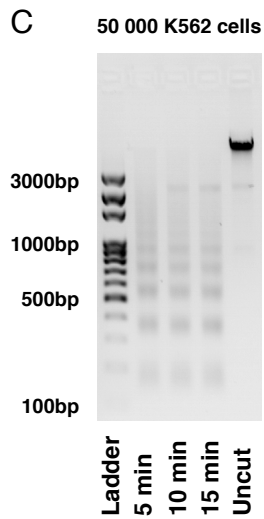

Supplement: S1 Fig — A Restriction of chromatin using 10 frequently cutting restriction endonucleases. B Combining more restriction endonucleases in a single reaction results in more efficient chromatin fragmentation. C Test of restriction enzyme 5-, 10- and 15-minute incubation times shows that 5 minutes is sufficient to fragment majority of the chromatin. (PDF) [file pone.0225801.s001.pdf]

A

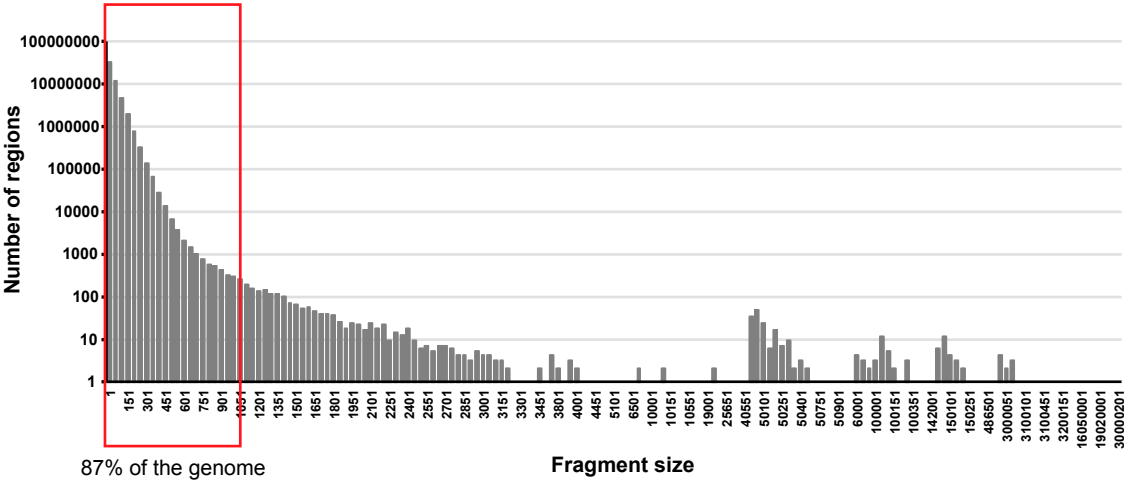

B

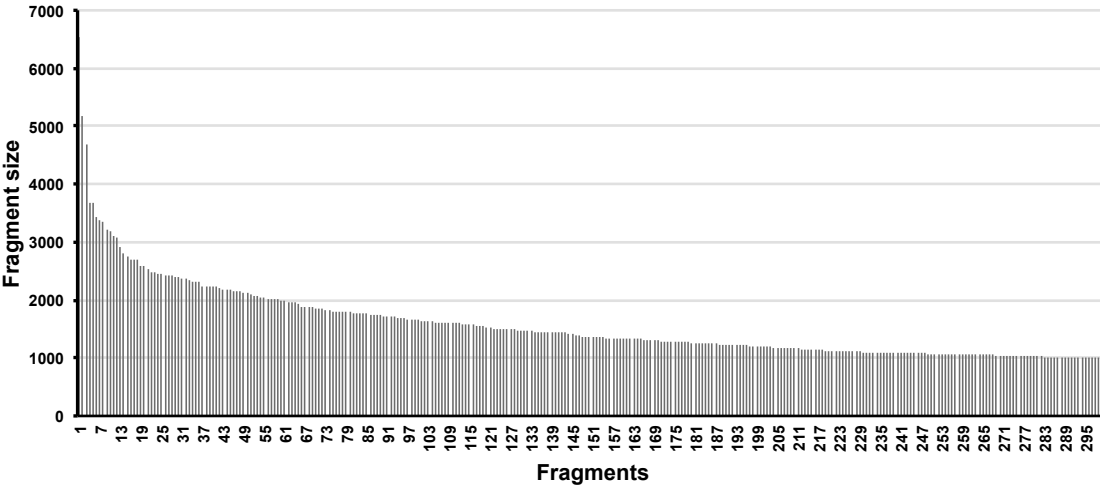

C

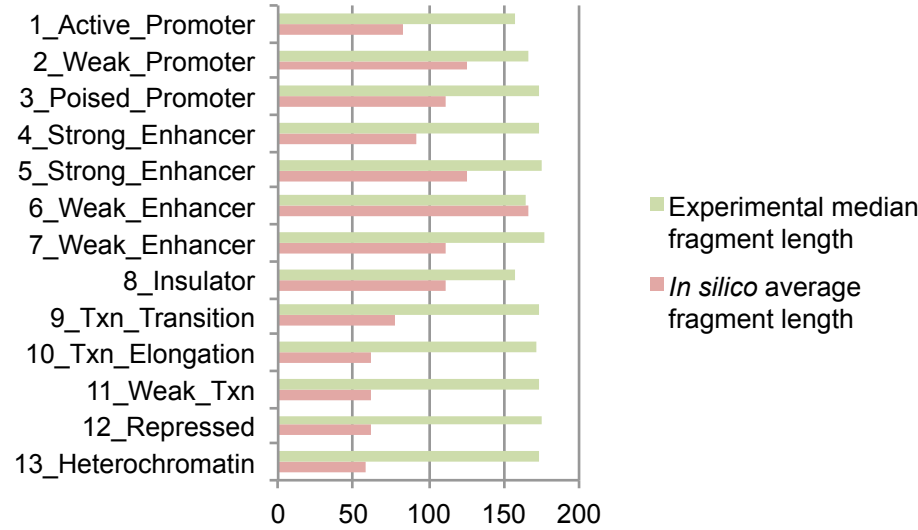

Supplement: S2 Fig — A Predicted fragment size distribution following cutting with AluI, SaqAI, MvaI and HinfI restriction endonucleases. B Size distribution of 299 fragments that remain larger than 1,000bp after in silico cutting with AluI, SaqAI, MvaI and HinfI restriction endonucleases. C In silico and experimental distribution of DNA fragment sizes in genomic regions with different chromatin states defined by chromHMM. (PDF) [file pone.0225801.s002.pdf]

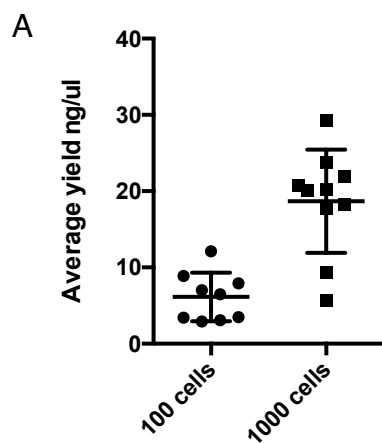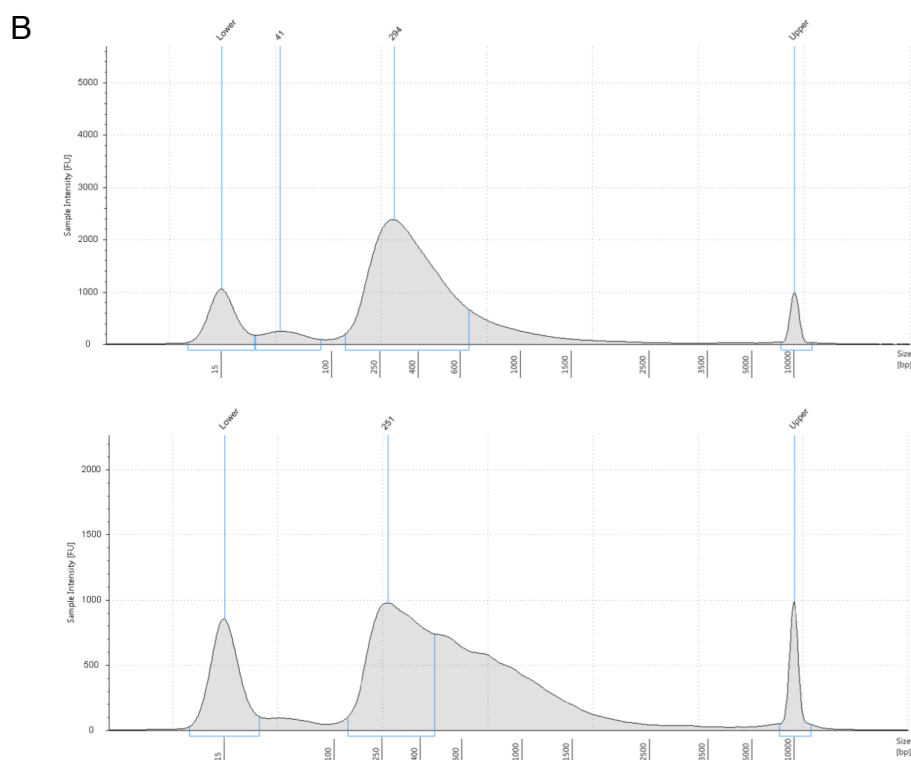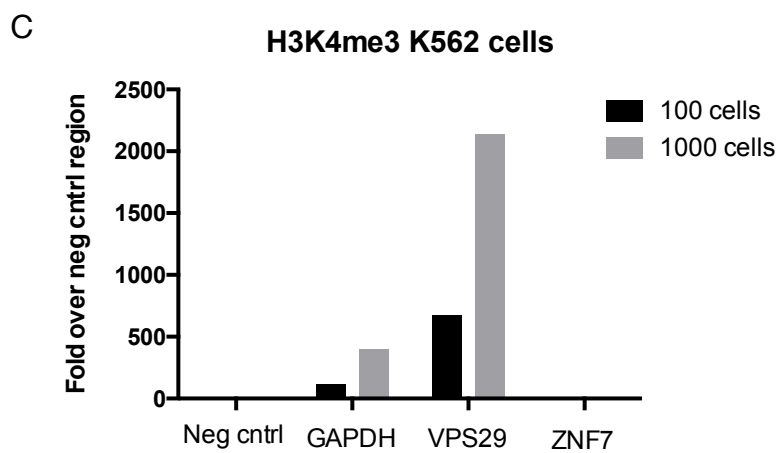

Supplement: S3 Fig — A Average yield of RAT-ChIP libraries from 100 and 1,000 cells after 16 rounds of PCR. B Example RAT-ChIP library analysed using TapeStation. C RAT-ChIP enrichments of H3K4me3 at the promoters of GAPDH, VPS29 and ZNF7 genes compared to negative control region using 100 and 1,000 cells. A representative experiment is shown. (PDF) [file pone.0225801.s003.pdf]

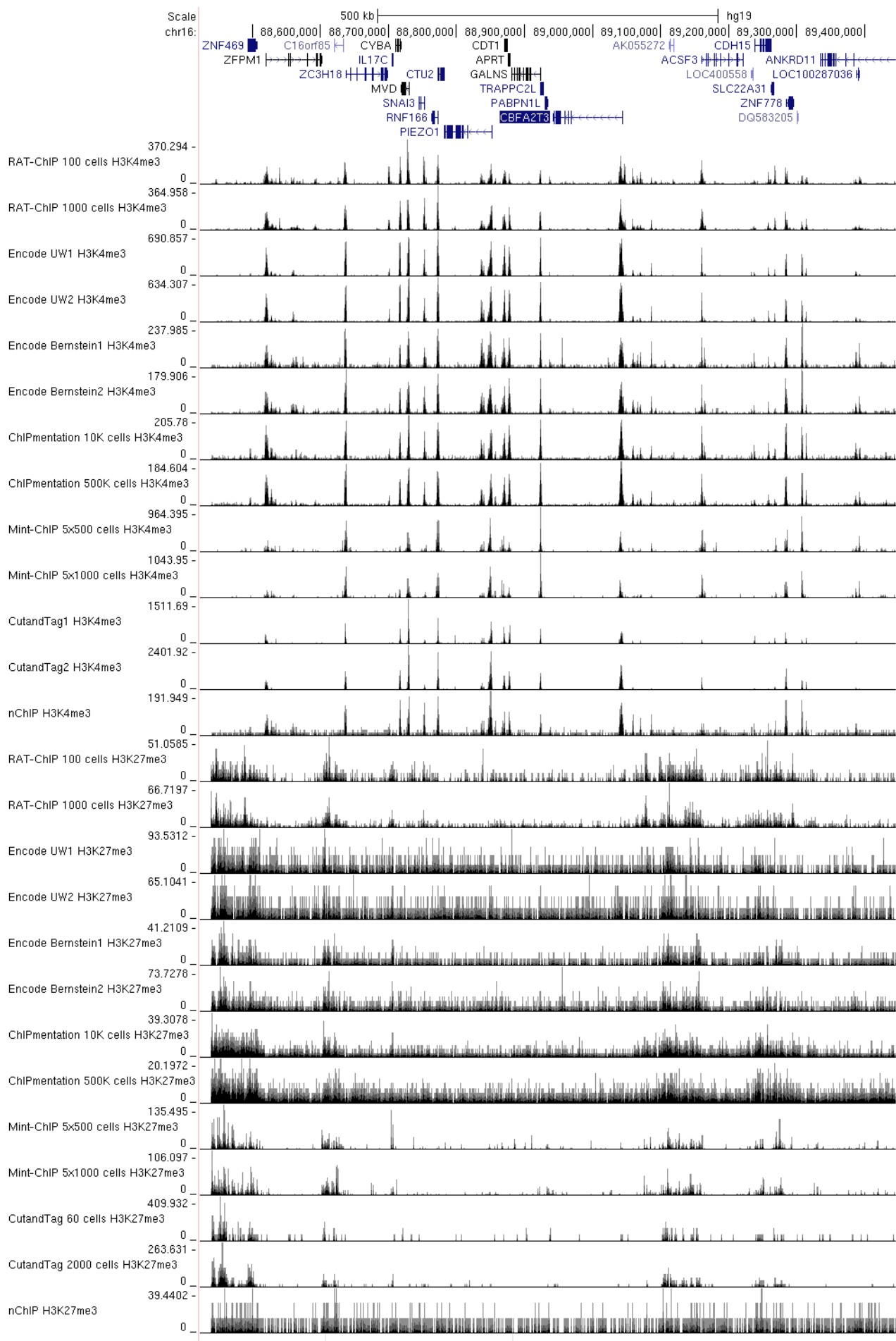

S4 Fig

Supplement: S4 Fig — Custom UCSC tracks of histone H3K4me3 and H3K27me3 profiles in ETO2 (CBFA2T3) gene locus in K562 cells to compared RAT-ChIP with other publicly available datasets. (PDF) [file pone.0225801.s004.pdf]

A Heatmaps of H3K4me3 profiles around the TSS

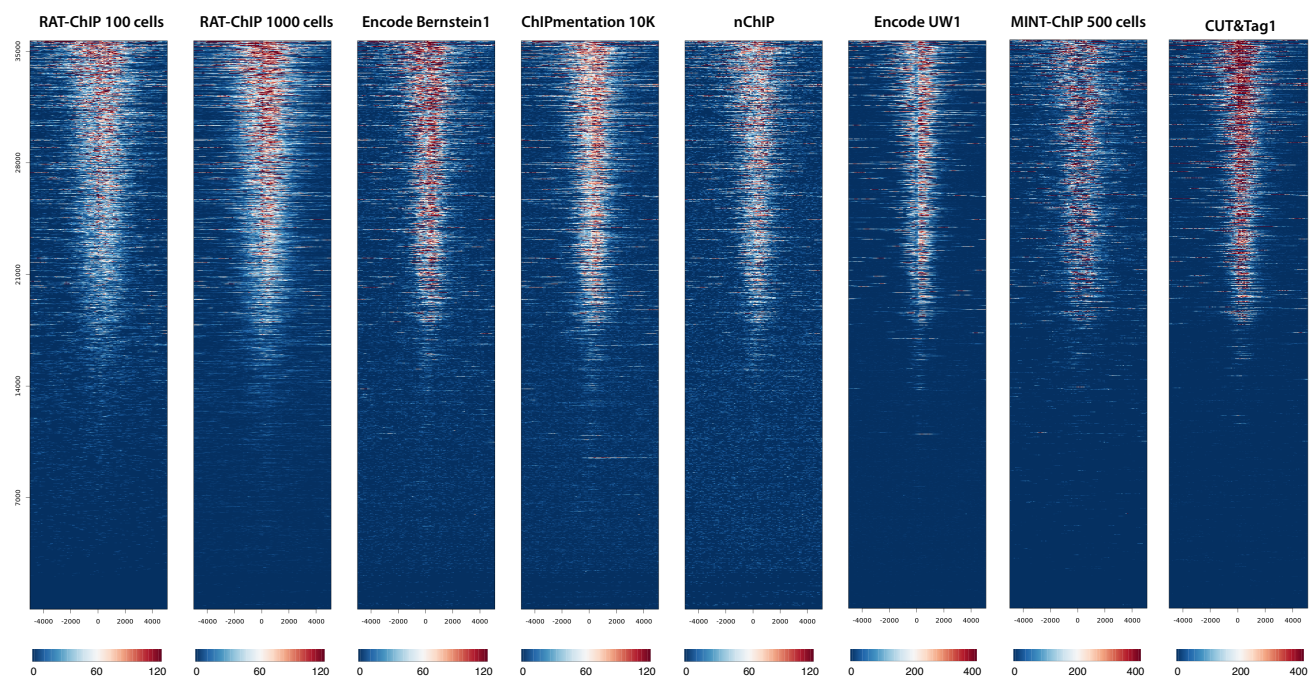

B Average H3K4me3 profiles around the TSS

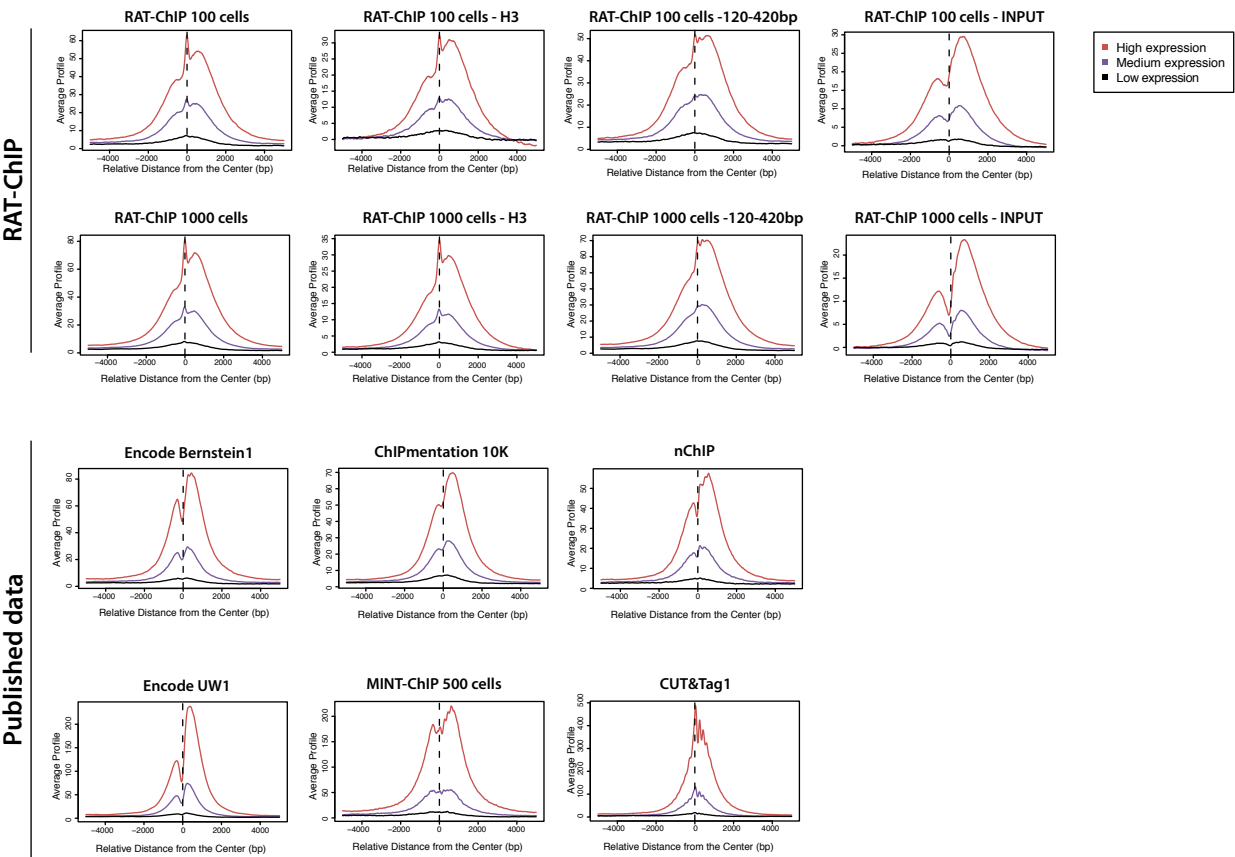

Supplement: S5 Fig — A Heatmaps of H3K4me3 signal in RAT-ChIP and published datasets in 10kb genomic regions around TSS of all NCBI RefSeq genes ranked by H3K4me3 signal intensity in RAT-ChIP 100 cell sample. B Average H3K4me3 signal intensities around 10kb region of NCBI RefSeq genes divided into 3 equally sized groups (high, medium and low expression) based on their expression levels using published RNA-seq experiment [47] RPKM values. RAT-ChIP data was additionally processed by subtracting either H3 or INPUT signal from the H3K4me3 signal or restricting analysis to reads with fragment sizes between 120 and 420 bp. (PDF) [file pone.0225801.s005.pdf]

Average H3K27me3 profiles around 6kb metagene bodies and 3kb up- and downstream of TSS and TES

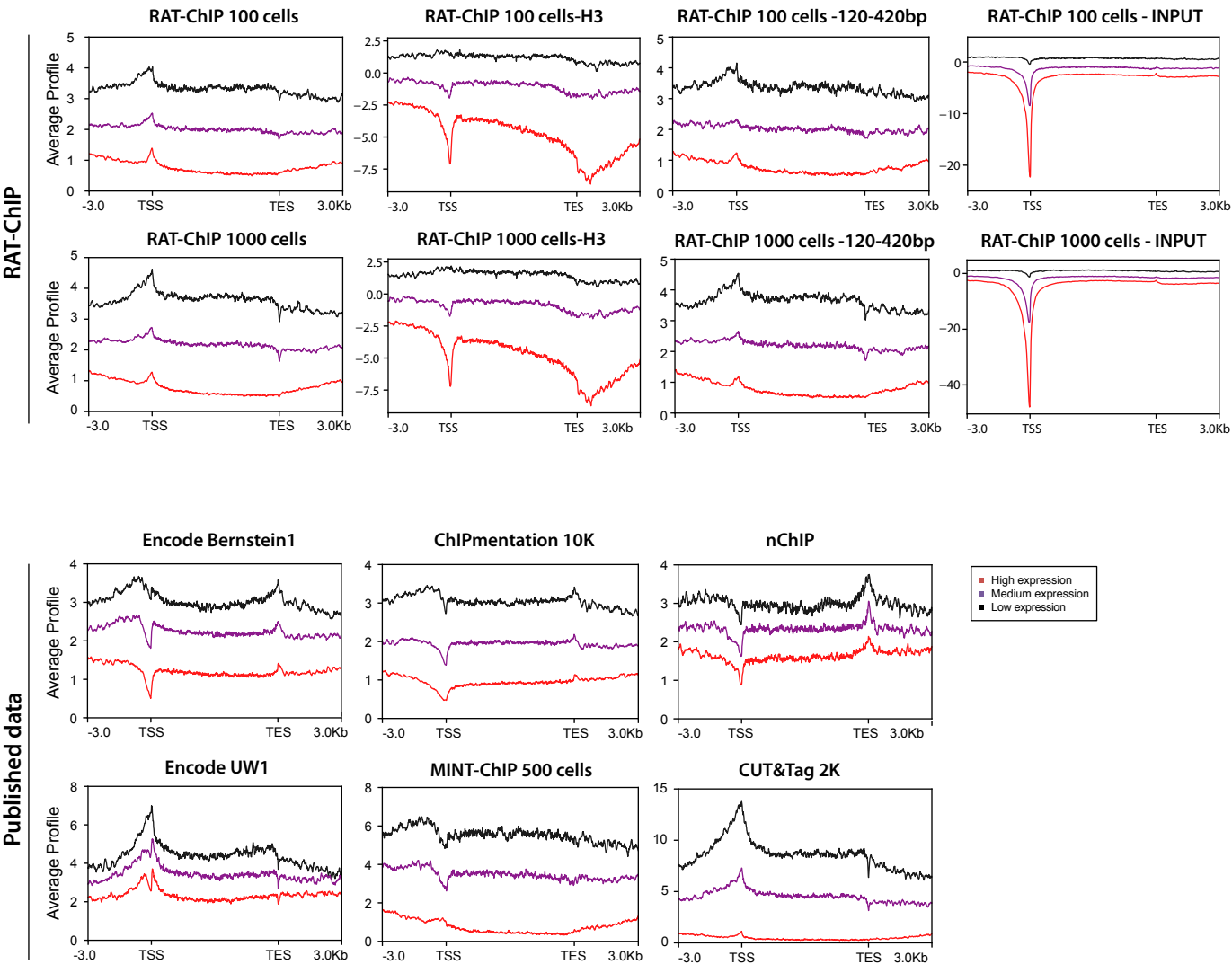

Supplement: S6 Fig — Average H3K27me3 signal intensities around 6kb metagene gene body and 3kb up and downstream of TSS (transcription start site) and TES (transcription end site) regions of NCBI RefSeq genes divided into 3 equally sized groups based on their expression levels (high, medium and low expression) using published RNA-seq experiment [47] RPKM values. RAT-ChIP data was additionally processed by subtracting either H3 or INPUT signal from the H3K4me3 signal or restricting analysis to reads with fragment sizes between 120 and 420 bp. (PDF) [file pone.0225801.s006.pdf]

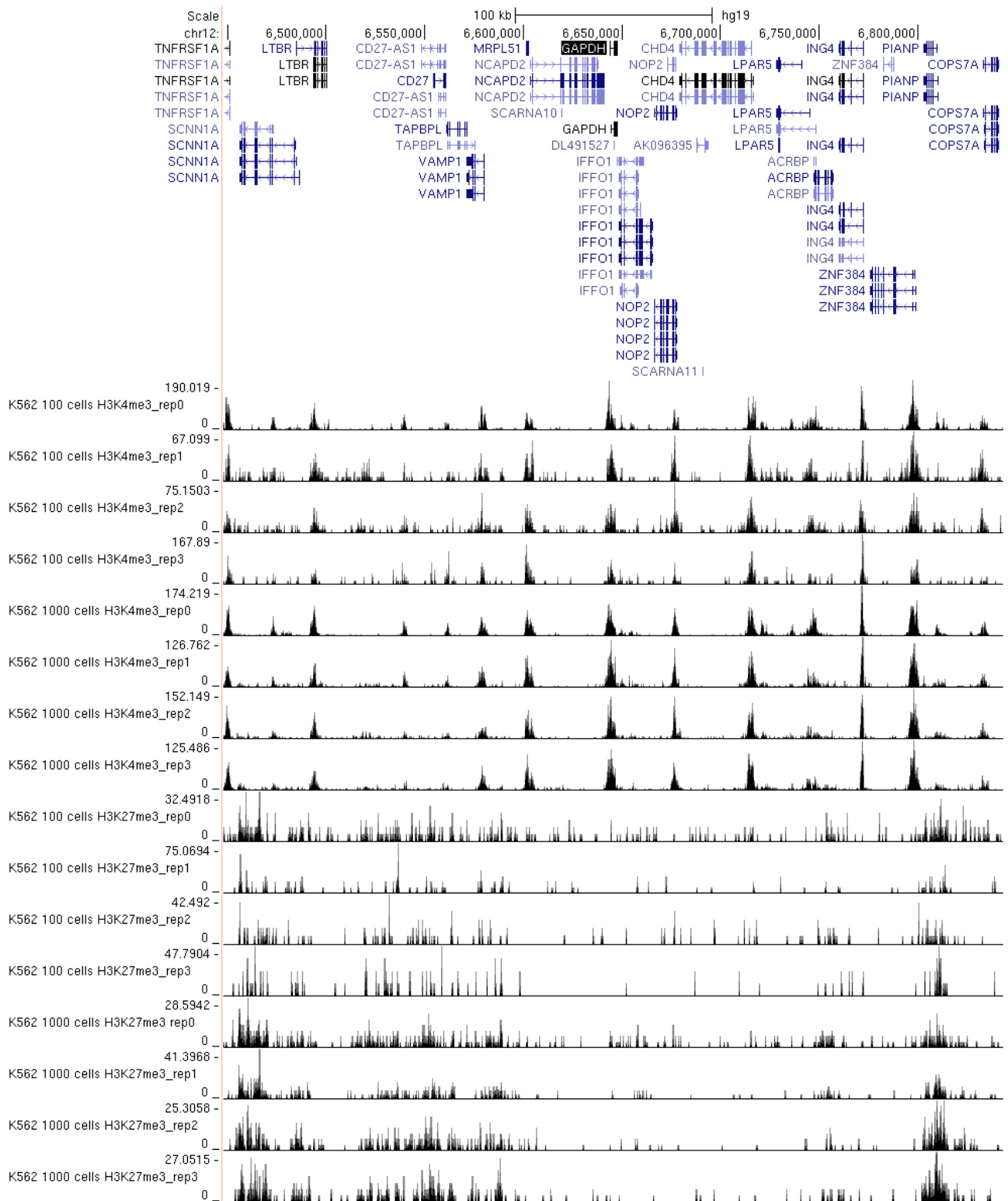

S7 Fig

Supplement: S7 Fig — Custom UCSC tracks of histone H3K4me3 and H3K27me3 profiles depicting 4 replicate RAT-ChIP experiments with 100 and 1000 cells in a genomic region surrounding GAPDH gene. (PDF) [file pone.0225801.s007.pdf]

## A H3K4me3

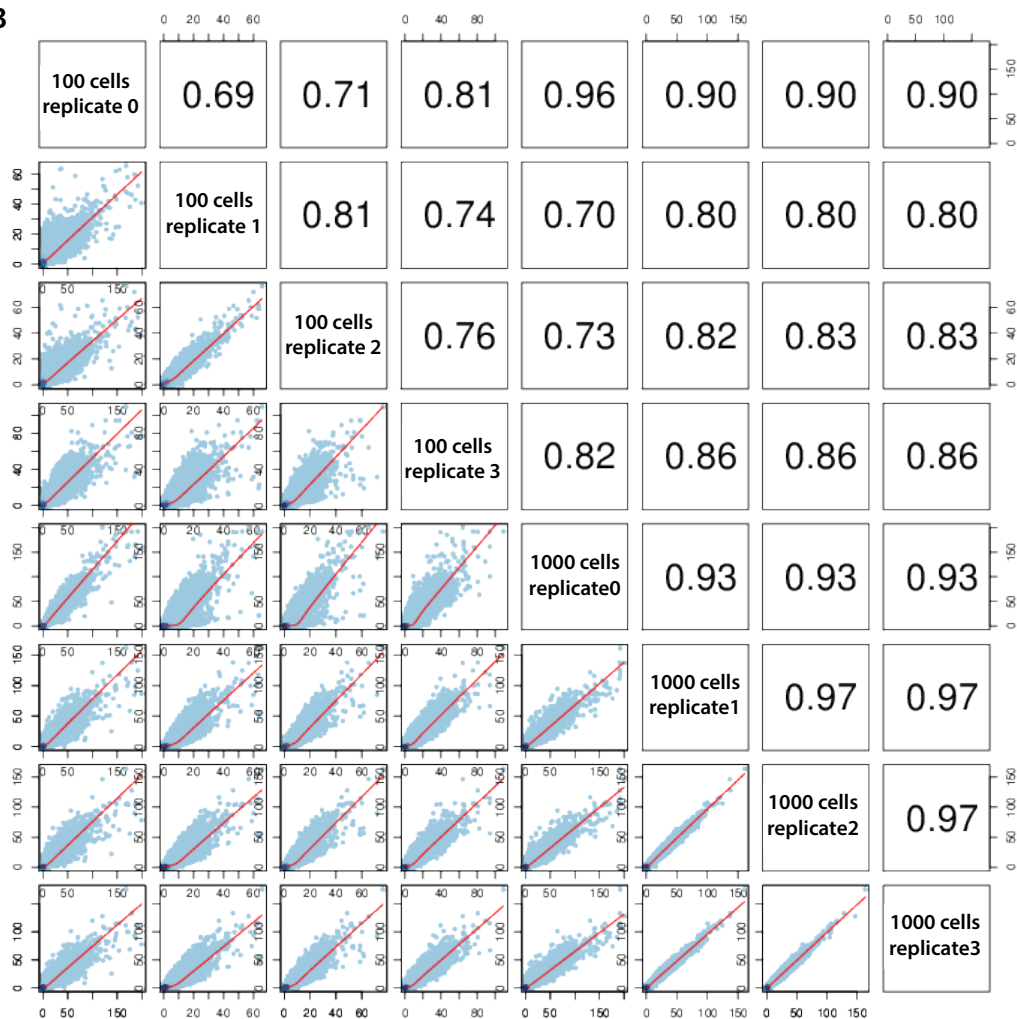

## B H3K27me3

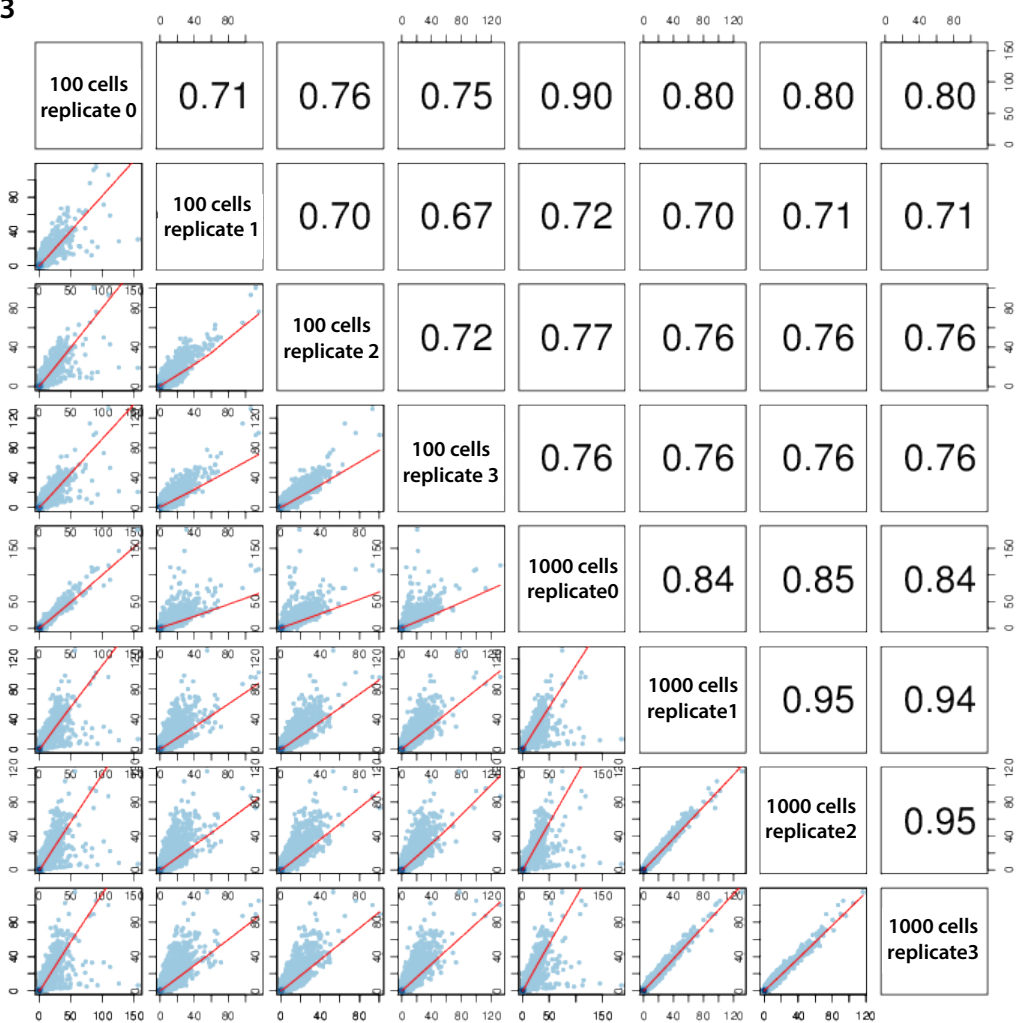

Supplement: S8 Fig — Scatterplots of pairwise comparisons of 8 replicate experiments (4 with 100 cells and 4 with 1000 cells) of histone H3K4me3 (A) and H3K27me3 (B) genome-wide signals in 5kb windows and corresponding Pearson correlation coefficients are shown. (PDF) [file pone.0225801.s008.pdf]

A

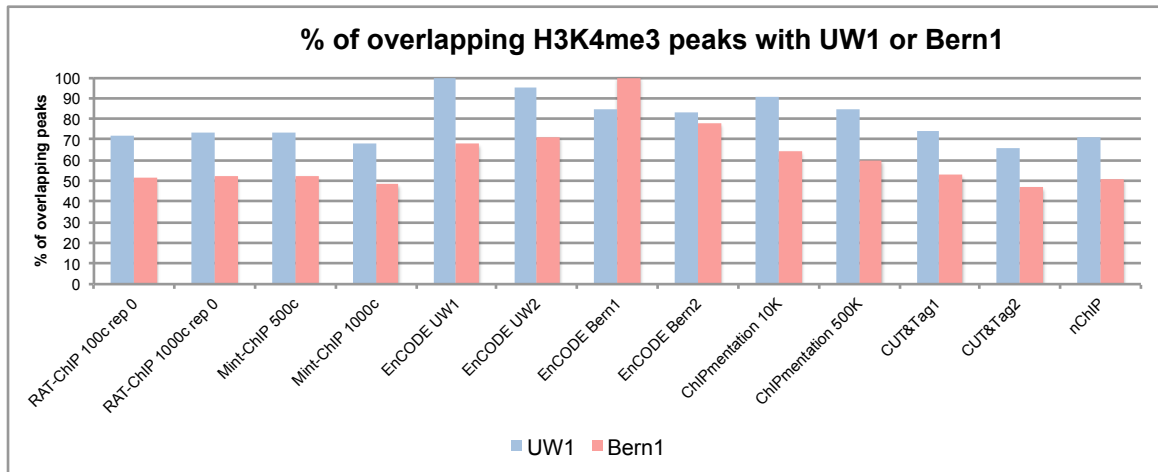

B

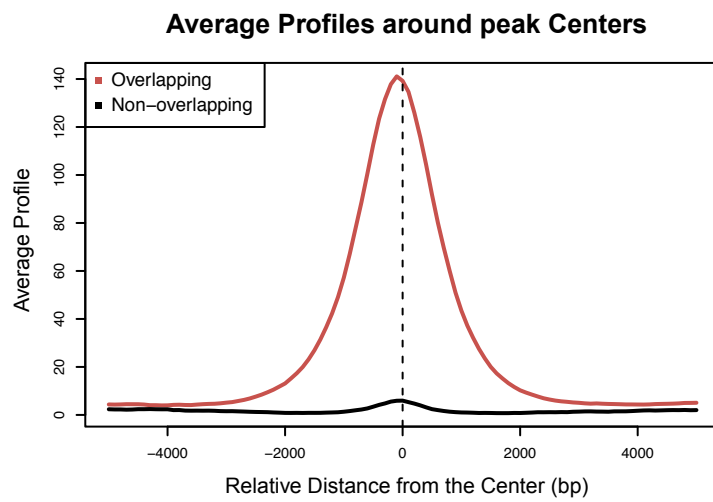

Supplement: S9 Fig — A Percentage of overlapping H3K4me3 SICER peaks of RAT-ChIP and published datasets using ENCODE UW1 or Bern1 peaks as a reference. B Average H3K4me3 profiles in UW1 dataset around peaks that overlap (red line) or do not overlap (black line) with RAT-ChIP data show that RAT-ChIP missed ENCODE peaks are low in enrichment. (PDF) [file pone.0225801.s009.pdf]

A

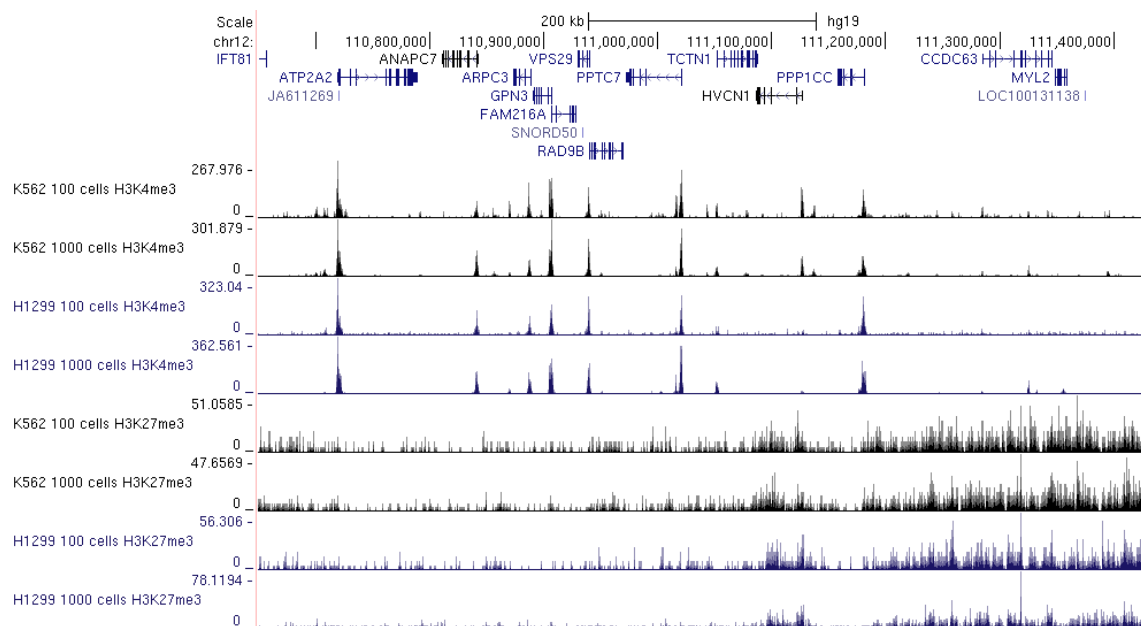

B

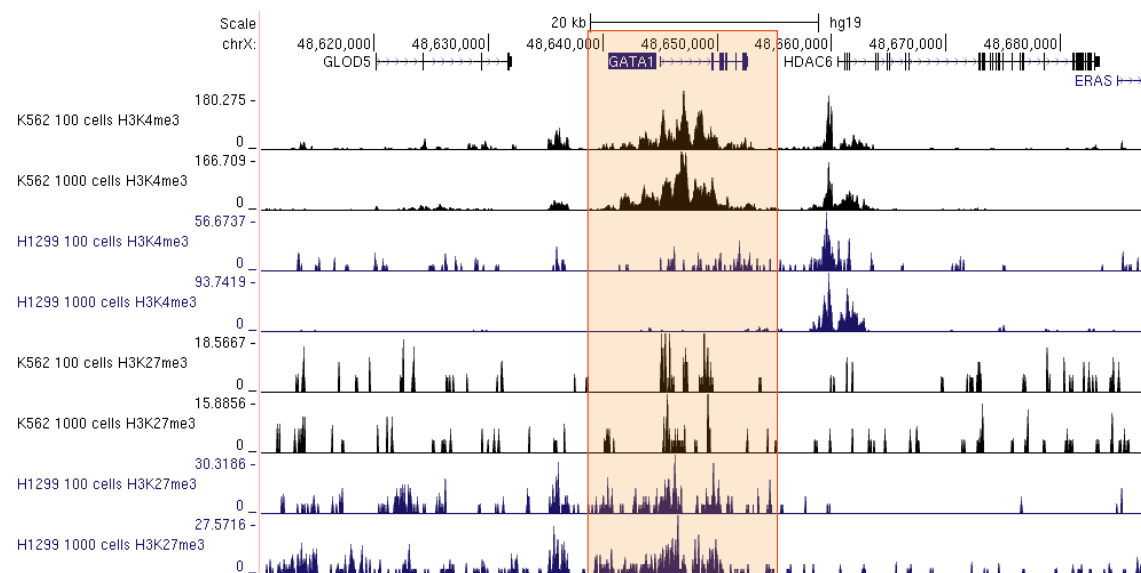

C

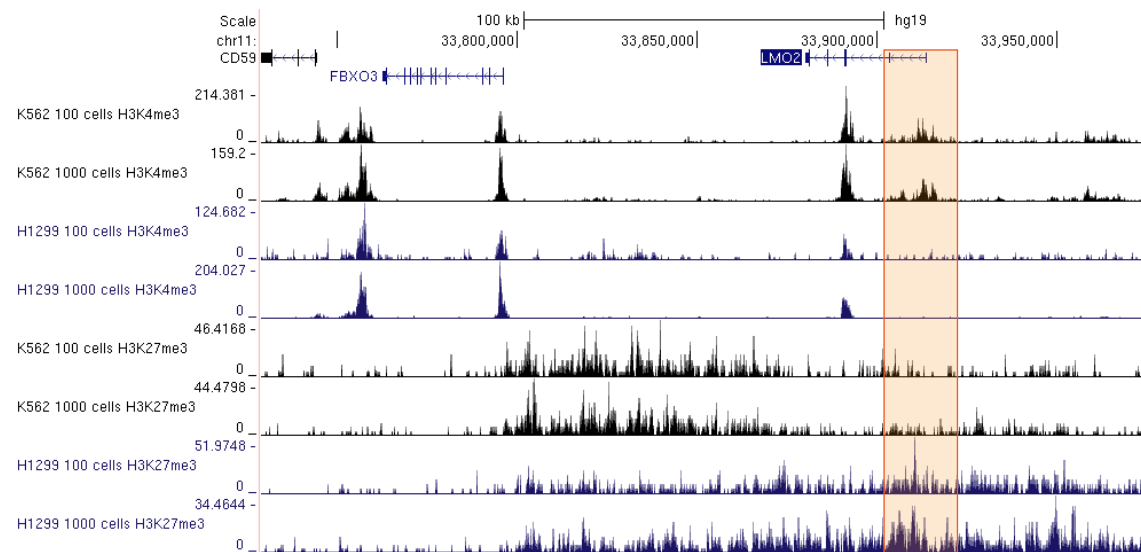

Supplement: S10 Fig — Custom UCSC tracks of histone H3K4me3 and H3K27me3 profiles in VPS29 (A), GATA1 (B) and LMO2 (C) gene loci in K562 cells compared to H1299 cells. (PDF) [file pone.0225801.s010.pdf]

A

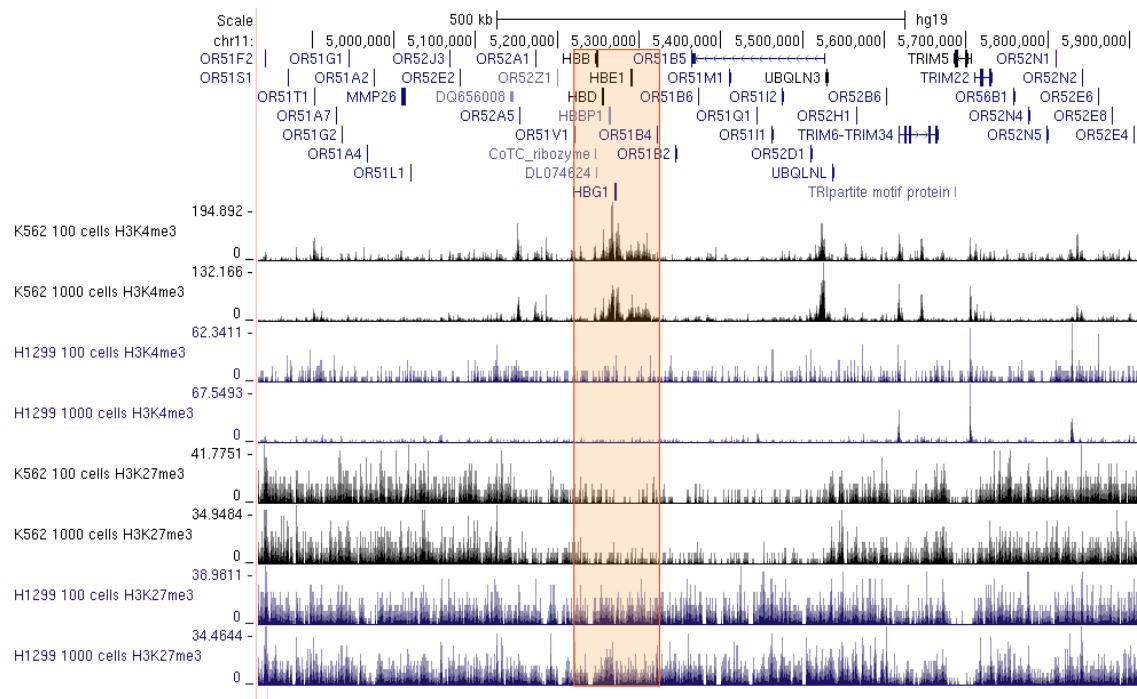

B

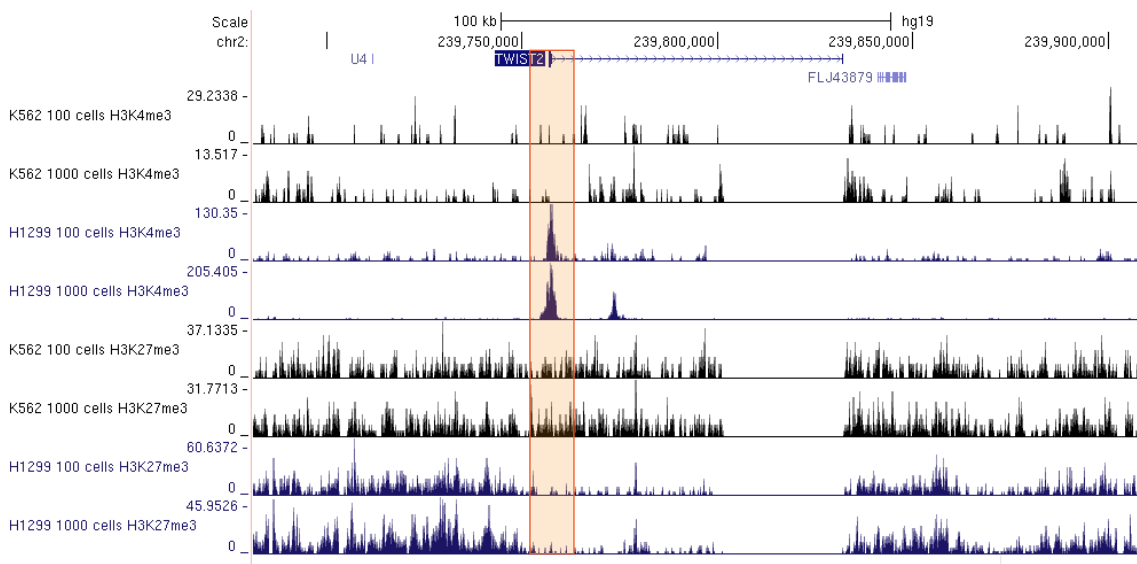

C

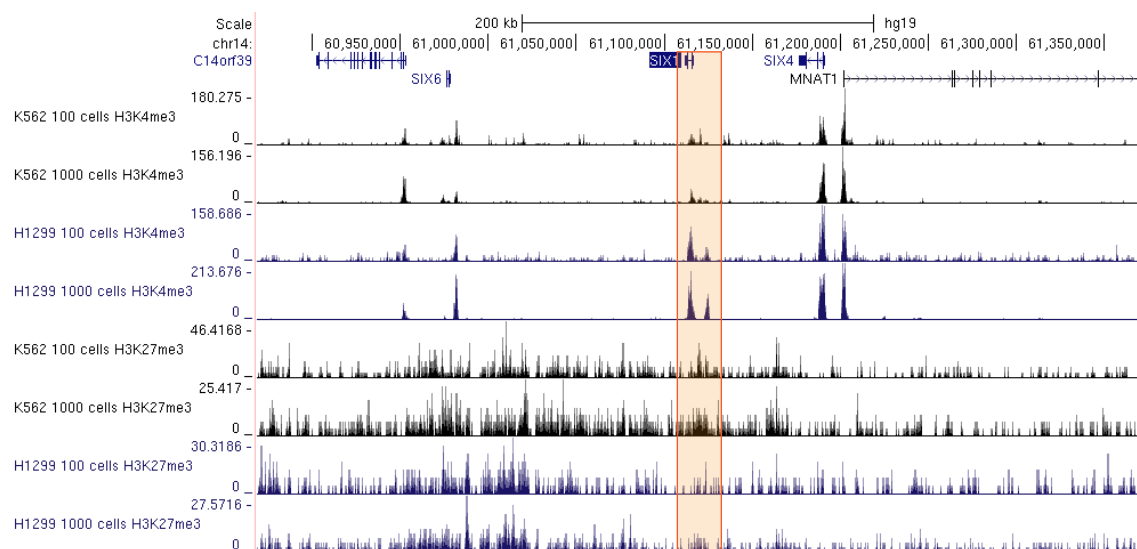

Supplement: S11 Fig — Custom UCSC tracks of histone H3K4me3 and H3K27me3 profiles in hemoglobin (A), TWIST2 (B) and SIX1 (C) gene loci in K562 cells compared to H1299 cells. (PDF) [file pone.0225801.s011.pdf]

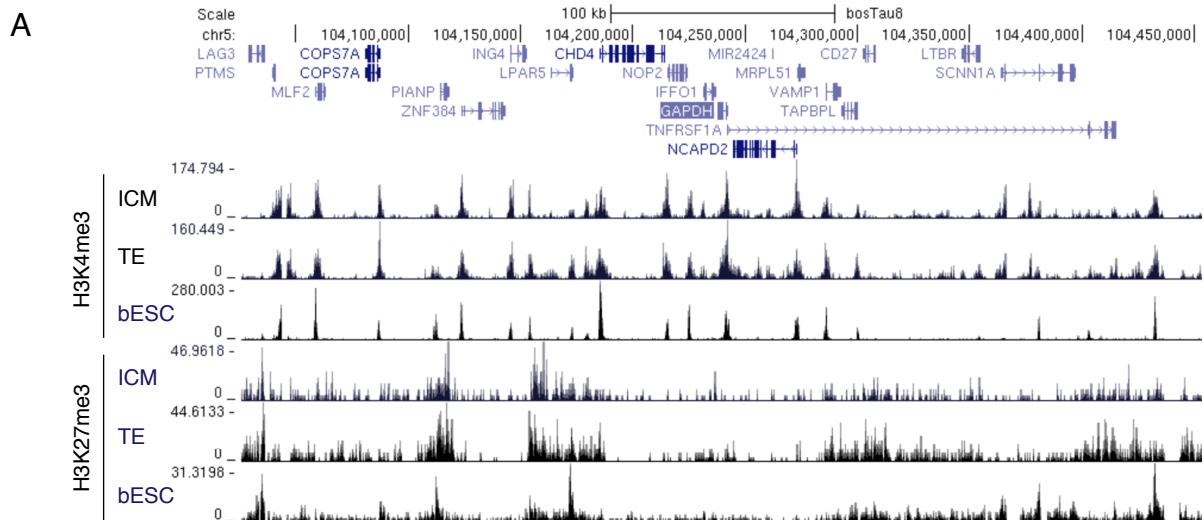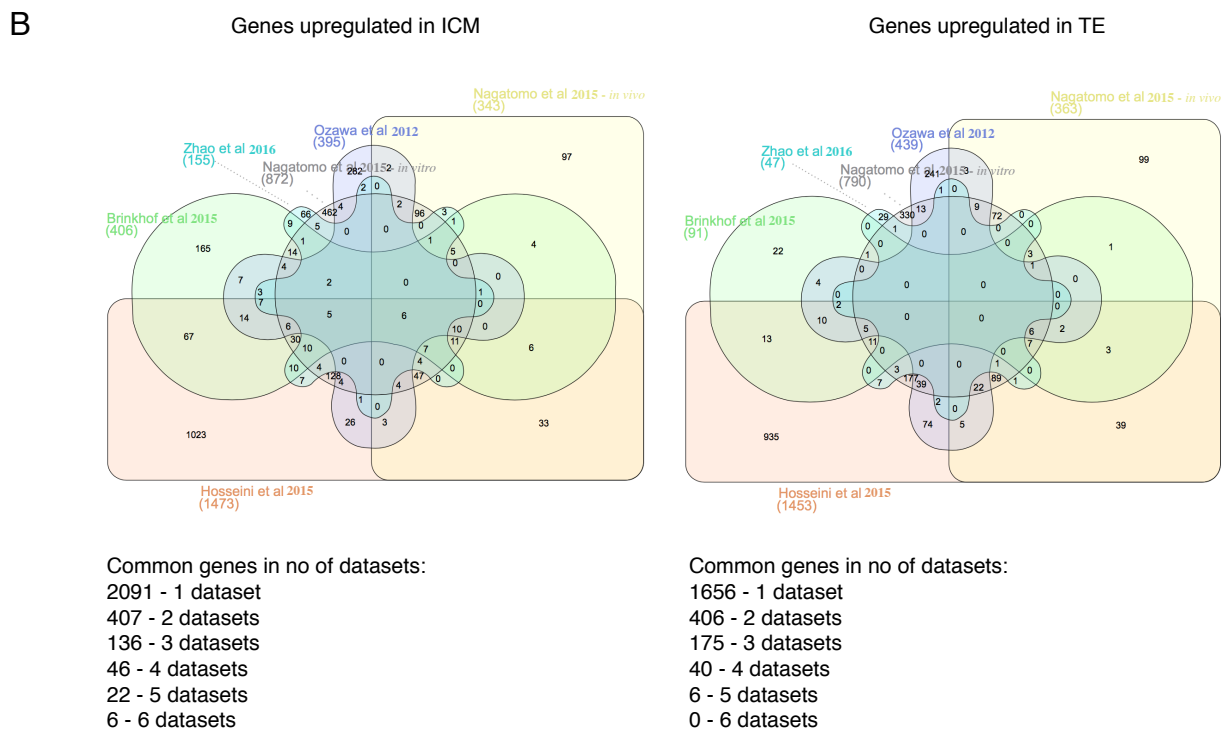

Supplement: S13 Fig — A Custom UCSC tracks of histone H3K4me3 and H3K27me3 RAT-ChIP profiles in GAPDH gene locus in ICM and TE of blastocyst stage embryos compared to published bESC data. B 6-way Venn diagram to show overlaps of genes from six published datasets that are upregulated in bovine blastocyst stage ICM (left) or TE (right). Below the Venn diagram is a summary of number of genes that overlap with a shown number of experiments. (PDF) [file pone.0225801.s013.pdf]

A

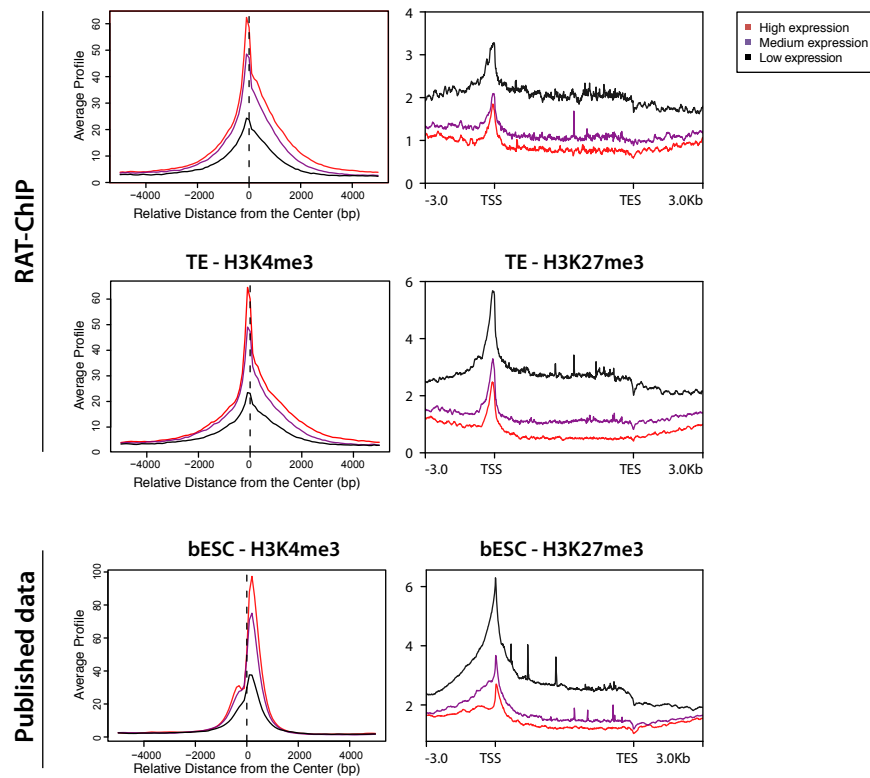

B

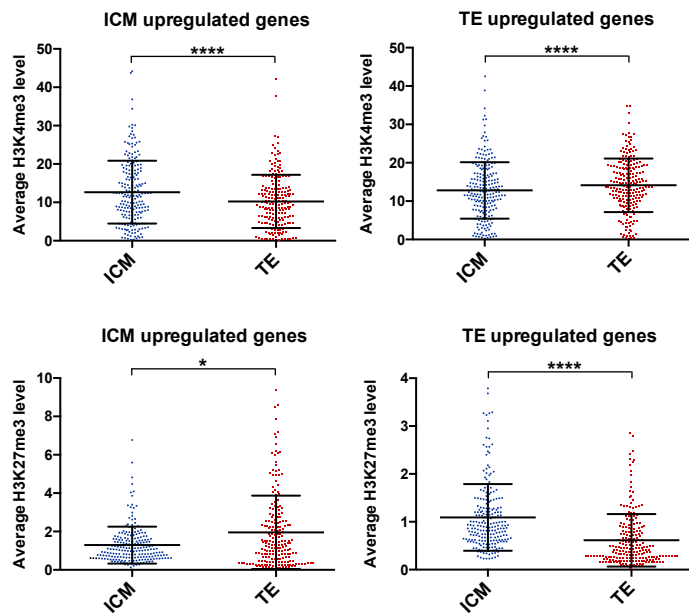

Supplement: S14 Fig — A Average H3K4me3 (10kb around TSS) and H3K27me3 (6kb metagene body and 3kb up and downstream of TSS and TES, respectively) signal in NCBI RefSeq gene regions divided into 3 equally sized groups (high, medium and low expression) based on their expression levels using published RNA-seq experiment [47] RPKM values in ICM or TE. Plots are shown for ICM, TE and published bESC data [48]. B Scatterplots of H3K4me3 (4kb region surrounding TSS) and H3K27me3 (gene body and 2kb upstream of TSS) average signal with mean and SD are shown for TE and ICM upregulated genes. Paired T-Test was used to calculate if average signals between pairs of corresponding gene regions (TE or ICM upregulated genes) in TE and ICM are significantly different. * p<0.05, **** p<0.0001 (PDF) [file pone.0225801.s014.pdf]

A

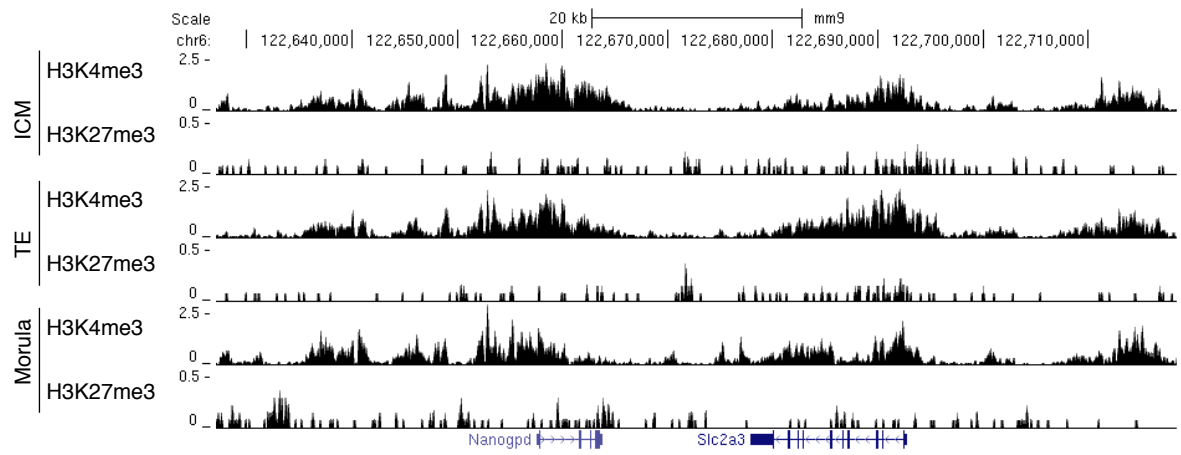

B

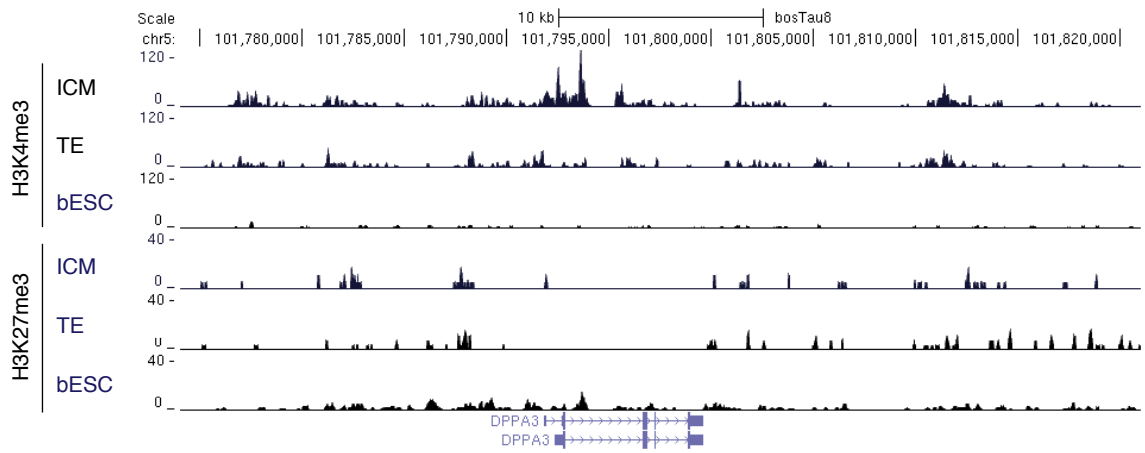

C

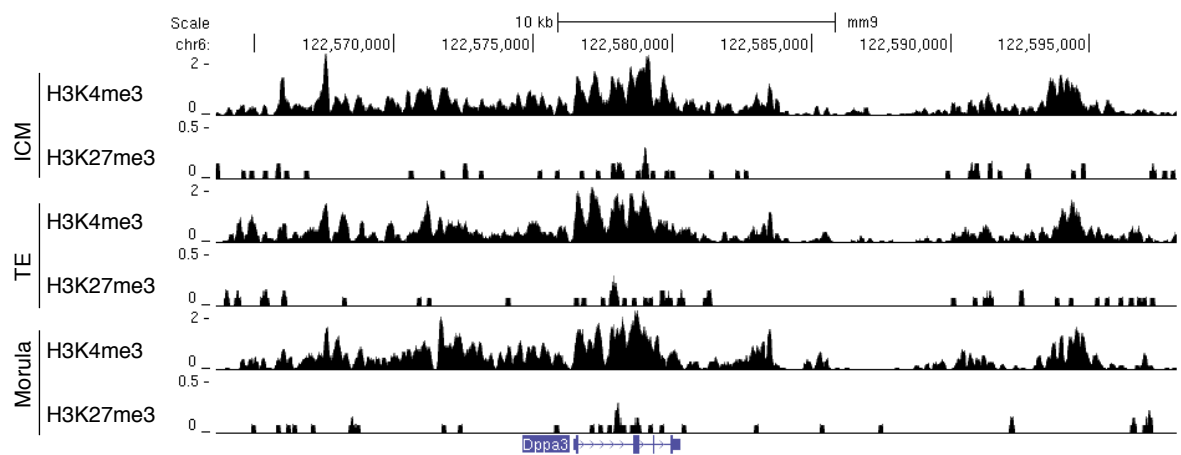

Supplement: S15 Fig — A Custom UCSC tracks of histone H3K4me3 and H3K27me3 profiles in Nanog gene locus in ICM and TE and morula of blastocyst stage mouse embryos [20]. B Custom UCSC tracks of histone H3K4me3 and H3K27me3 RAT-ChIP profiles in DPPA3 gene locus in ICM and TE of blastocyst stage embryos compared to published bESC data [48]. C Custom UCSC tracks of histone H3K4me3 and H3K27me3 profiles in Dppa3 gene locus in ICM and TE and morula of blastocyst stage mouse embryos [20]. (PDF) [file pone.0225801.s015.pdf]
