# Supplementary material for: Genome-wide histone modification profiling of inner cell mass and trophectoderm of bovine blastocysts by RAT-ChIP
Source: PLoS One. 2019 Nov 25;14(11):e0225801. doi: 10.1371/journal.pone.0225801 (PMC6876874; doi:10.1371/journal.pone.0225801)
Supplement: S12 Fig — A Heatmap of histone H3K27me3 signal in K562 and H1299 cells in 10kb regions centered around TSS of 300 genes with either cell type specific or common signal. B Enriched biological processes GO terms of GREAT analysis of differentially enriched regions between K562 and H1299 cells. (PDF) [file pone.0225801.s012.pdf]

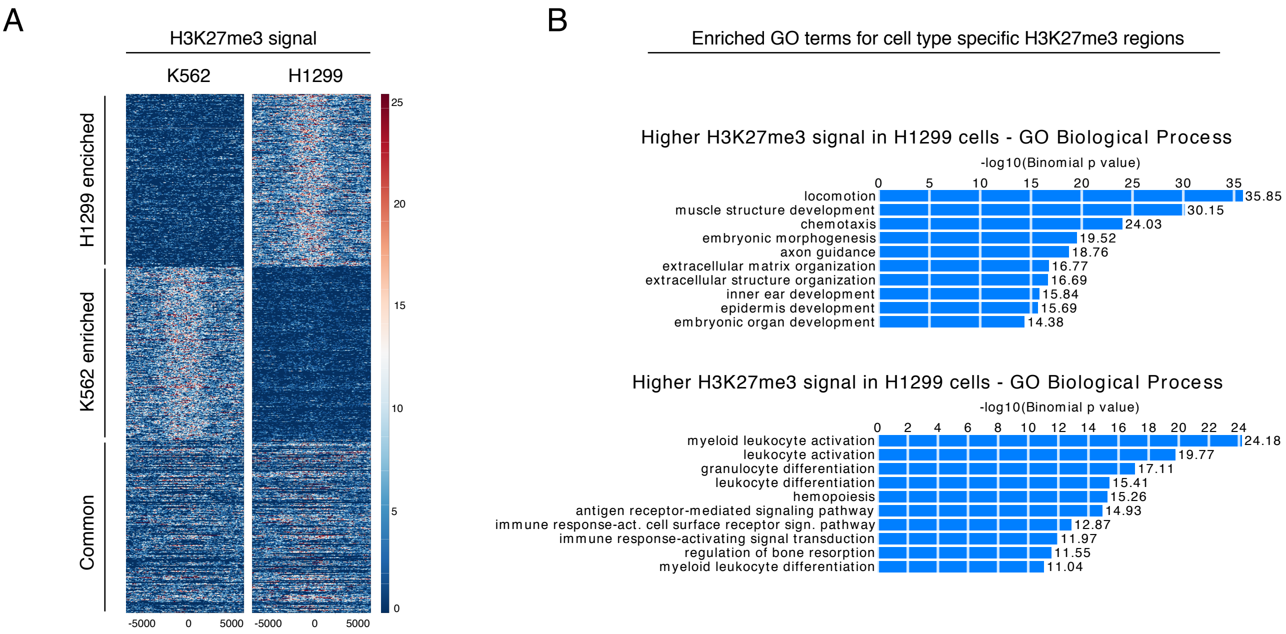
A
H3K27me3 signal
K562
H1299
H1299 enciched
K562 enriched
Common
25
20
15
10
5
0
-5000
0
5000
-5000
0
5000
B
Enriched GO terms for cell type specific H3K27me3 regions
Higher H3K27me3 signal in H1299 cells - GO Biological Process
-log10(Binomial p value)
0
5
10
15
20
25
30
35
locomotion 35.85
muscle structure development 30.15
chemotaxis 24.03
embryonic morphogenesis 19.52
axon guidance 18.76
extracellular matrix organization 16.77
extracellular structure organization 16.69
inner ear development 15.84
epidermis development 15.69
embryonic organ development 14.38
Higher H3K27me3 signal in H1299 cells - GO Biological Process
-log10(Binomial p value)
0
2
4
6
8
10
12
14
16
18
20
22
24
myeloid leukocyte activation 24.18
leukocyte activation 19.77
granulocyte differentiation 17.11
leukocyte differentiation 15.41
hemopoiesis 15.26
antigen receptor-mediated signaling pathway 14.93
immune response-act. cell surface receptor sign. pathway 12.87
immune response-activating signal transduction 11.97
regulation of bone resorption 11.55
myeloid leukocyte differentiation 11.04

S12 Fig
