# Supplementary material for: Genome-wide histone modification profiling of inner cell mass and trophectoderm of bovine blastocysts by RAT-ChIP
Source: PLoS One. 2019 Nov 25;14(11):e0225801. doi: 10.1371/journal.pone.0225801 (PMC6876874; doi:10.1371/journal.pone.0225801)
Supplement: S1 File — (PDF) [file pone.0225801.s016.pdf]

S1 File. RAT-ChIP-seq protocol

The RAT-ChIP-seq protocol has seven major steps:
1. Bind antibodies to protein G Dynabeads
2. Cell preparation for IP
3. Chromatin immunoprecipitation
4. On beads tagmentation
5. PCR for sequencing library construction
6. PCR purification
7. Library quality control and sequencing

The amounts given in the protocol below are for a single immunoprecipitation (IP). When working with multiple samples, please scale the volumes accordingly. For simultaneous processing of multiple samples please use multichannel pipets and 0.2ml 8-strip PCR tubes.

Step 1. Bind antibodies to protein G Dynabeads

- Pipet 1μl of protein G Dynabeads per IP into standard 0.2ml eppendorf tube containing 50 μl of IP buffer at RT.
- Capture the magnetic beads by 1 min incubation on a magnetic stand (Diagenode) and careful removal of the buffer.
- Take beads up in, 2 times the volume of beads (2μl per IP), IP buffer. Add 0.25μg of corresponding antibody per IP and incubate 2 h at RT with end-over end mixing (30 rpm).
- Wash the beads 2 times with 50 μl of IP buffer at RT by gently pipetting the beads 10 times up and down.
- Suspend the beads in the original amount of IP buffer (1μl per IP).

NB! We recommend preparing one sample for IP with general histone H3 antibody, that can be used as an input. Process the H3 sample identical to the other samples.

Step 2. Cell preparation for IP

- Collect your cells of interest and count density using heamocytometer.
- Centrifuge 1000 g for 5 min, remove supernatant by pipeting and take the pellet up in PBS at desired density (for example 100 cells or 1000 cells) per 0.5μl.
- Prepare 10μl of nuclear lysis/restriction buffer by combining:

0.5μl of PIC (protease inhibitor cocktail) (Roche)
1μl 10x FastDigest buffer (Thermo Fisher Scientific)
0.5μl of restriction enzyme mix (AluI, SaqAI, MvaI, HinfI - mixed in equal volumes)
8μl of 2x nuclear lysis buffer

- Mix 0.5µl cells with 0.5µl of nuclear lysis/restriction buffer and incubate 10 min on ice and 5 min at 37°C.

- Put samples back to ice and add:
1µl of 0.2%TritonX-100/0.2% NaDOC solution

- Incubate 10 min on ice and wortex for 30 sec.

Step 3. Chromatin immunoprecipitation

- To prepare the samples for immunoprecipitation add:

8µl of IP buffer
1µl of antibody bound Dynabeads

- Perform IP 4h at 4°C with end-over end mixing (30 rpm).
- Wash 3 times with 100ul of low salt buffer, 3 times with 100ul of high salt buffer, 1 time with 100µl of IP buffer and 1 time with Tris HCl pH7.4 by gently pipetting the beads 10 times up and down. Capture the magnetic beads by 1min incubation on a magnetic stand (Diagenode).
- After adding Tris HCl pH7.4, suspend the beads and carry them over to a new 0.2ml tube to reduce background.

NB! Be careful when removing Tris HCl pH7.4 buffer during the last wash, as the beads tend to come loose from the magnet!

Step 4. On beads tagmentation

- Prepare tagging mix by combining (scale according to number of reactions):

2.5µl 2x Tagmentation buffer from Nextera DNA Library preparation kit
2.5µl mQ
0.5µl of Tn5 transposase

- Add 5µl of the mix to the magnetic beads, resuspend by pipetting and perform tagmentation 1min at 37°C.

- Wash once with 50µl of low salt buffer and once with TE.

Step 5. PCR for sequencing library construction

NB! Please choose appropriate primers for the samples that you want to sequence simultaneously. Ad1_noMX primer is always the same. Ad2 primers have different barcodes that during data processing will be used to assign the reads to a correct sample.

- Prepare PCR mix (scale according to the number of reactions):

10µl of NEBNext High-Fidelity 2X PCR Master Mix
5µl mQ
2.5µl Ad1_noMX primer (5uM)

- Add 17.5µl of mix directly to washed beads and finally add 2.5µl of corresponding Ad2.X primer (5µM) and do 16 cycles of PCR using the following program:

1. 72°C 5 min,
2. 98°C 2 min,
3. 98°C 10 sec,
4. 63°C 10 sec,
5. 72°C 1 min,
6. repeat steps 3-5 15 times
7. hold at 4°C

Step 6. PCR purification

- After PCR put samples to magnetic rack and take 20µl of supernatant to a clean 0.2ml tube.
- Add 20µl of mQ to and 40µl of Agencourt RNA XP magnetic beads (1:1).
- Incubate 10 min at RT.
- Wash 2 times with 100ul of 70% EtOH .
- Dry beads 5 min at RT.
- Elute with 10µl of Tris HCl pH7.4 by pipetting and remove supernatant after 1 min incubation on magnetic rack.

Step 7. Library quality control and sequencing

Quantify the library (by Nanodrop, Qubit, TapeStation etc.) and perform sequencing using Illumina platform.

Materials

Dynabeads Protein G for Immunoprecipitation (Thermo Fisher)
Catalog #: 10004D

Nextera DNA library preparation kit 24 samples (Illumina)
Catalog #: FC-121-1030

NEBNext High-Fidelity 2X PCR Master Mix - 250 rxns (New England Biolabs)
Catalog #: M0541L

Agencourt RNAClean XP Magnetic Beads (Beckman Coulter)
Catalog #: A63987

cOmplete™ Protease Inhibitor Cocktail (Roche)
Catalog #: 4693116001

FastDigest Buffer (10X) (Thermo Fisher) - Catalog #: B64
FastDigest AluI (Thermo Fisher) - Catalog #: FD0014
FastDigest MvaI (Thermo Fisher) - Catalog #: FD0554
FastDigest HinfI (Thermo Fisher) - Catalog #: FD0804
FastDigest SaqAI (Thermo Fisher) - Catalog #: FD2174

Buffers

2x Nuclei preparation buffer:
- 20 mM Tris HCl pH 7.4
- 20 mM NaCl
- 6 mM $MgCl_2$
- 0.2% IGEPAL CA-630 (NP-40)

Complete Immunoprecipitation buffer (IP)
- 20 mM Tris-HCl pH 8.0
- 2 mM EDTA
- 150 mM NaCl
- 0.1% Triton X-100
- 1x Protease inhibitor cocktail (Roche)

Low Salt Wash buffer
- 20 mM Tris-HCl pH 8.0
- 2 mM EDTA
- 150 mM NaCl
- 1% Triton X-100

High Salt Wash buffer
- 20 mM Tris-HCl pH 8.0
- 2 mM EDTA
- 500 mM NaCl
- 1% Triton X-100
- 0.1% SDS

TE buffer
- 10 mM Tris-HCl pH 8.0
- 1 mM EDTA

PCR primer sequences for sequencing library generation:

Ad1_noMX:	AATGATACGGCGACCACCGAGATCTACACTCGTCGGCAGCGTCAGATGTG
Ad2.1_TAAGGCGA	CAAGCAGAAGACGGCATACGAGATTCGCCTTAGTCTCGTGGGCTCGGAGATGT
Ad2.2_CGTACTAG	CAAGCAGAAGACGGCATACGAGATCTAGTACGGTCTCGTGGGCTCGGAGATGT
Ad2.3_AGGCAGAA	CAAGCAGAAGACGGCATACGAGATTTCTGCCTGTCTCGTGGGCTCGGAGATGT
Ad2.4_TCCTGAGC	CAAGCAGAAGACGGCATACGAGATGCTCAGGAGTCTCGTGGGCTCGGAGATGT
Ad2.5_GGACTCCT	CAAGCAGAAGACGGCATACGAGATAGGAGTCCGTCTCGTGGGCTCGGAGATGT
Ad2.6_TAGGCATG	CAAGCAGAAGACGGCATACGAGATCATGCCTAGTCTCGTGGGCTCGGAGATGT
Ad2.7_CTCTCTAC	CAAGCAGAAGACGGCATACGAGATGTAGAGAGGTCTCGTGGGCTCGGAGATGT
Ad2.8_CAGAGAGG	CAAGCAGAAGACGGCATACGAGATCCTCTCTGGTCTCGTGGGCTCGGAGATGT
Ad2.9_GCTACGCT	CAAGCAGAAGACGGCATACGAGATAGCGTAGCGTCTCGTGGGCTCGGAGATGT
Ad2.10_CGAGGCTG	CAAGCAGAAGACGGCATACGAGATCAGCCTCGGTCTCGTGGGCTCGGAGATGT
Ad2.11_AAGAGGCA	CAAGCAGAAGACGGCATACGAGATTGCCTCTTGTCTCGTGGGCTCGGAGATGT
Ad2.12_GTAGAGGA	CAAGCAGAAGACGGCATACGAGATTCCTCTACGTCTCGTGGGCTCGGAGATGT
Ad2.13_GTCGTGAT	CAAGCAGAAGACGGCATACGAGATATCACGACGTCTCGTGGGCTCGGAGATGT
Ad2.14_ACCACTGT	CAAGCAGAAGACGGCATACGAGATACAGTGGTGTCTCGTGGGCTCGGAGATGT
Ad2.15_TGGATCTG	CAAGCAGAAGACGGCATACGAGATCAGATCCAGTCTCGTGGGCTCGGAGATGT
Ad2.16_CCGTTTGT	CAAGCAGAAGACGGCATACGAGATACAAACGGGTCTCGTGGGCTCGGAGATGT
Ad2.17_TGCTGGGT	CAAGCAGAAGACGGCATACGAGATACCCAGCAGTCTCGTGGGCTCGGAGATGT
Ad2.18_GAGGGGTT	CAAGCAGAAGACGGCATACGAGATAACCCCTCGTCTCGTGGGCTCGGAGATGT
Ad2.19_AGGTTGGG	CAAGCAGAAGACGGCATACGAGATCCCAACCTGTCTCGTGGGCTCGGAGATGT
Ad2.20_GTGTGGTG	CAAGCAGAAGACGGCATACGAGATCACCACACGTCTCGTGGGCTCGGAGATGT
Ad2.21_TGGGTTTC	CAAGCAGAAGACGGCATACGAGATGAAACCCAGTCTCGTGGGCTCGGAGATGT
Ad2.22_TGGTCACA	CAAGCAGAAGACGGCATACGAGATTGTGACCAGTCTCGTGGGCTCGGAGATGT
Ad2.23_TTGACCCT	CAAGCAGAAGACGGCATACGAGATAGGGTCAAGTCTCGTGGGCTCGGAGATGT
Ad2.24_CCACTCCT	CAAGCAGAAGACGGCATACGAGATAGGAGTGGGTCTCGTGGGCTCGGAGATGT
